# Organizational changes in diabetic foot care practices for patients at low and moderate risk after implementing a comprehensive foot care program in Alberta, Canada

**DOI:** 10.1186/s13047-020-00393-0

**Published:** 2020-05-19

**Authors:** Catherine B. Chan, Kathy Dmytruk, Michele Labbie, Petra O’Connell

**Affiliations:** 1 Diabetes, Obesity and Nutrition Strategic Clinical Network, Alberta Health Services, 10101 Southport Road, Calgary, Alberta T2W 1S7 Canada; 2 Department of Agricultural, Food and Nutritional Sciences, University of Alberta, 4-126 Li Ka Shing Centre, Edmonton, Alberta T6G 2E1 Canada; 3 Department of Physiology, University of Alberta, 7-55 Medical Sciences Building, Edmonton, Alberta T6G 2H7 Canada; 4 WestView Health Centre, 4405 South Park Drive, Stony Plain, Alberta T7Z 2M7 Canada

**Keywords:** Diabetes, Foot ulcer, Clinical pathway, Prevention, Screening, Wounds and injuries, High risk foot team

## Abstract

**Background:**

Neuropathy and vasculopathy can lead to costly and debilitating complications in people with diabetes. The purpose of this study was to evaluate, at an organizational level, uptake of practices included in a diabetic foot care clinical pathway and associated resources. This research focused on patients at low and moderate risk in Alberta, Canada between 2014 to 2019.

**Methods:**

Serial surveys (2014, 2019) of practices related to screening and care of the feet of people with diabetes. Surveys were administered using a combination of targeted and snowball sampling in order to assess the impact of the clinical pathway first implemented in 2015. The pathway focused on screening, assessment and referral of patients from primary care. High-risk foot teams (HRFT) were established at six sites to provide increased access to specialty care. Comparative statistics were performed to assess differences in footcare practices between 2014 and 2019 using two-tailed Fisher’s exact test or Chi-square test.

**Results:**

Respondents (*n* = 104, 2014 and *n* = 75, 2019) included personnel from primary health care, home care and long-term care, acute and emergency care, specialty clinics, diabetes-specific programs and private contractors. The proportion of primary care and home care/long-term care (HC/LTC) sites providing screening increased significantly (*p* < 0.05). A significant increase in the proportion of sites providing assessment for patients designated as moderate risk also increased from 35% (34 out of 96 sites) to 55% (36 out of 65 sites) (*p* < 0.05), particularly with respect to vascular assessment, and the proportion of sites reporting appropriate follow-up intervals according to the pathway recommendation was also improved.

**Conclusion:**

Provision of a clinical pathway for diabetic foot care along with education and resources led to increased screening in primary care and HC/LTC settings in Alberta, Canada. HRFT provided primary healthcare providers with an important option for referral and also provided increased expertise for procedures such as vascular assessment for patients with moderate risk of ulceration. This comprehensive model has the potential to reduce progression of foot problems and overall health services utilization. Further analyses of outcomes such as incident lower limb amputation and long-term cost-effectiveness of pathway implementation are underway.

## Introduction

Diabetic foot ulcer (DFU) is a severe diabetes complication often leading to lower limb amputations. In Canada in 2011, more than 25,000 prevalent cases resulted in 6036 amputations with a cost per prevalent case of $21,371 CAD [1]. International studies have found the 5-year mortality rate after new-onset ulceration to be 43–55%, and as high as 74% for people with diabetes who had a lower limb amputation [2]. DFU and lower limb amputations in people with diabetes are potentially preventable in 75% [3] and 50–85% of cases, respectively, through screening, early treatment and better self-care practices [4]. Clinical pathway development, clinician training and education, and provision of procedural support tools are all shown to increase foot screening and improve treatment [5, 6]. Patient education is also recommended as important to reduce risk of diabetic foot complications [7]. Efforts to prevent DFU in patients identified at moderate to high risk, have a strong possibility of cost-effectiveness [8] probably through reduction of emergency department or inpatient admissions, which are increased by 3-fold in patients with DFU compared with other ambulatory clinic cases [9]. Provision of care by multi-disciplinary teams to prevent and manage foot wounds is recommended [10–12], as is having a structured approach to assessing clinical and metabolic parameters and assigning risk categories to patients [13]. In Queensland, Australia, implementation of state-wide protocols for diabetic foot care reduced major amputation by 45% [14].

In the jurisdiction of Alberta, Canada, diabetic foot disease was identified in 2013 as a priority for action by the Diabetes, Obesity and Nutrition (DON) Strategic Clinical Network™ (SCN) of Alberta Health Services (AHS) [15]. As part of the pathway development process, the DON SCN™ conducted a survey with both quantitative and qualitative elements in order to understand existing foot care services across Alberta. The unpublished survey results identified low screening capacity, barriers to referral, and lack of standardized approaches were supported by self-reported data from people with diabetes. This showed that self-care practices and screening frequencies [16] were not in accordance with Diabetes Canada 2013 guidelines for foot care [4], a problem also reported for Canada as a whole [17]. In 2014, the DON SCN™ began the process of implementing a new care pathway for diabetic foot care focusing on DFU prevention through screening and creation of multidisciplinary High Risk Foot Teams (HRFT) to facilitate referrals.

The literature shows that integrated care models for secondary prevention of DFU or LLA are medically and cost-effective [8, 18]. However, there is a paucity of literature on efficacious and cost-effective integrated care models for primary prevention of DFU [3, 19]. Approaches using multi-disciplinary teams appear to be efficacious but overall quality of the studies is not optimal [10]. Based on guidelines of Diabetes Canada [4], Wounds Canada [20] and the Registered Nurses Association of Ontario (RNAO) [21], the Alberta Health Services (AHS) diabetes foot care pathway was developed. It encompasses elements of assessment, planning, implementation and evaluation of foot care strategies for patients using a patient-centred approach and addressing appropriate treatment of comorbidities [22]. The pathway also acted upon RNAO recommendations to include professional education and guidelines to cover policy and organizational considerations. It also provided resources to monitor and support quality improvement, develop an effective referral strategy, advocate for client services that promote offloading of pressure to prevent re-ulceration [21].

In 2015, the DON SCN™ conducted a pilot project at three sites followed by scale and spread across Alberta. The pathway utilizes HRFT that provide a foot protection service and support primary care. The primary care service performs the initial screening, care for low risk patients, and continued care for patients with healed ulcers once released from specialty care. A screening instrument and modules to support patient education have also been made available along with education on their utilization. HRFT also provide referral to surgeons, providers of specialty footwear and offloading devices, and other supports for patients [23].

The aim of current study was to assess the impact of implementing a diabetes foot care clinical pathway and associated resources and training on organizational changes in diabetic foot care practices in 2019 compared with the baseline state in 2014 in Alberta, Canada. This research focused on organizational capacity for foot screening and care of low-moderate risk patients. In comparison with responses in 2014 we predict enhanced diabetic foot care practices, particularly at sites with access to HRFT.

## Research methods

### Study design

Serial cross-sectional assessments of organizational practices related to foot care of people with diabetes.

### Study setting

Health care sites that play a role in the care of diabetic foot screening and treatment across the continuum in Alberta were identified. These were primary health care, home care and long-term care, acute and emergency care, specialty clinics, diabetes-specific programs and private contractors. Sites that actively participated in implementation of the diabetes foot care pathway, as well as those that did not were included in the survey. The 2019 survey was conducted between February and April. The 2014 survey was conducted in September.

### Participants

A convenience sample of representatives of the identified health care sites were recruited, including physicians, nurse practitioners, registered nurses, licensed practical nurses, foot care nurses, orthopedic surgeons, physio- or occupational therapists, dietitians or pharmacists, diabetes educators, clinic managers or any practitioner who had contacted the DON SCN™ about foot care, including private service providers (e.g., foot care nurses, podiatrists). In 2014, the extent of sites and individuals participating in care of patients with diabetic foot disease was less well known; thus snowball sampling initiated with a small number of key contacts was used to distribute the survey as widely as possible. The total number of sites approached to participate was undetermined. In 2019, a list of potential participants were developed based on the previous respondents to the survey in 2014. In addition, we invited attendees at four AHS-sponsored foot care symposia held in 2017 and 2018, and utilized personal knowledge to invite other individuals involved in diabetic foot care as well . This greatly expanded the potential participant list compared with 2014. In both 2014 and 2019, potential participants were contacted by email with an invitation, the study information and a hyperlink to the survey. Potential participants received two email reminders to complete the survey following the initial invitation. Waiver of consent was granted for the 2014 survey by the Research Ethics Board of the University of Alberta. In 2019, informed consent was implied by completion of the survey.

### Intervention

A literature review to identify best practice, informed the process of developing the intervention, including the care pathway and ancillary resources. In addition, the Project Lead (author KD) consulted experts in the United Kingdom (UK) regarding their pathway development process and implementation of foot protection teams, which assess and treat patients at risk of, or presenting with DFU [24]. The Canadian Agency for Drugs and Technology and Health organized and facilitated a meeting between four provinces, including Alberta, to discuss current foot and wound care practices. Following this, Alberta and New Brunswick teams embarked on pathway development and the implementation of “high risk foot teams” (foot protection teams) similar to what had been implemented in the UK. The New Brunswick team allowed the DON SCN™ to leverage the work they had started in the development of a diabetes foot care clinical pathway. The DON SCN™ struck a working group composed of diabetes educators, primary care physicians and nurses, home care, wound care nurses and nurse practitioners and foot care nurses (licensed practical nurses (LPNs)), including authors KD and ML. The working group developed The Alberta Diabetes Foot Care Clinical Pathway tools and resource toolkit (Supplementary Materials). It was guided by the tools and resources developed by New Brunswick (diabetic foot screening tool, health provider guide, referral guidelines, patient handouts on low, moderate and high risk foot problems) and Canadian guidelines [20, 21, 25]. All tools and resources in the toolkit were vetted with and approved by a provincial steering committee. Members of the steering committee consisted of podiatric surgeons, orthopedic surgeons, dermatologist, physiatrists, endocrinologist, primary care physician, wound care nurses and nurse practitioners, and foot care nurses.

The diabetes foot care pathway focussed on three key elements: screening, assessment and referral pathways that could be customized depending on site characteristics. Implementation in primary care and home care/long-term care (HC/LTC) settings focused on screening and assessment supported by a novel screening tool, a risk assessment triage referral form, training from a nurse with specialized wound care training, videos and other supports. Goals for screening and assessment included increased competency of primary care providers to accurately categorize a person with diabetes as low, moderate, high or urgent risk for foot ulceration and amputation, to increase screening rates, and to provide patient education using resources developed by the DON SCN™. Within primary health care, appropriate actions taken on patients with moderate risk feet (i.e., having conditions such as callus, structural deformities or loss of protective sensation) have the potential to reduce risk of future ulceration. This included educating patients on the importance of routine self-care activities. Moderate risk assessment included inspection for skin and nail abnormalities, assessment of structural deformities (e.g., bunions, hammertoes), assessing whether special footwear was necessary, and evaluating vascular and peripheral nervous function.

To address barriers to timely referrals of high or urgent risk patients, in part related to Alberta’s large rural and remote population, HRFT were constituted in strategic locations. Depending on the site, HRFT were led by physicians, nurse practitioners, nurses, licensed practical nurses, or occupational therapists and included at least one other provider, preferably one member holding prescribing privileges for medications, diagnostic testing and referral to specialty care. Such multidisciplinary teams are recommended in various guidelines and are often known as foot protection teams [4, 11, 21, 26, 27]. In the Alberta model, HRFT assess and treat patients at risk of a DFU and those with an ulcer present. HRFTs have wound management and debridement expertise, lower leg vascular assessment training, can prescribe footwear through a provincially funded resource for medical aids, Alberta Aids to Daily Living (AADL). AADL is funded by Alberta Health and is mandated to help Albertans with a long-term disability, chronic or terminal illness to pay for basic medical equipment and supplies. However, only specified healthcare professional may prescribe AADL services. Skilled deployment of more complex testing and treatment modalities (e.g. debridement) is essential in preventing ulcers from progressing. Some HRFTs have the ability to perform skin and nail care (nail trimming/callus management) as an adjunct service. Other HRFTs do not provide this service; patients can be referred to a community podiatrist, foot care nurse, or other medically trained provider in the community. HRFT make formal linkages with other referral services such as diabetes clinics, vascular laboratories, occupational therapists or orthotists. HRFT could refer to specialists such as community podiatrists (private practitioners) or podiatric surgeons (focusing on wound management and limb preservation) as outlined in the pathway. In addition to the DON SCN™-sponsored sites, other sites throughout Alberta have developed similar multidisciplinary models of care in primary care or outpatient settings.

In addition to the specific pathway intervention, the DON SCN™ also created a number of tools to support screening, assessment and HRFT, which were freely available via a website [23] (Supplemental Materials). Awareness of the pathway was achieved through newsletters, word of mouth, symposiums, and presentations at meetings and conferences.

For purposes of comparative analyses, 2014 data were considered as “usual care” controls compared with 2019 post-intervention data as a whole. The 2019 data was also stratified by whether sites accessed HRFT and their practices compared to each other.

### Intervention delivery

Between 2014 and 2019, 8 sites (2 North Zone, 2 Edmonton Zone, 2 Central Zone, 1 Calgary Zone, 2 South Zone) received training from the DON SCN™ personnel in all elements of the intervention including use of the clinical pathway and associated resources (toolkit) and established HRFT. Additional education consisted of training in performing a diabetes foot screen, using the pathway tools to navigate the patient to the most appropriate resource and follow-up timelines depending on risk level. This training was provided to anyone requesting it, mainly in primary care settings but also community pharmacists and indigenous healthcare providers. Of 41 Primary Care Networks in Alberta, 14 received in person training while education was provided to personnel in most First Nations health centres in Alberta via videoconference. The wound care nurse provided in-person training, with education on how to use the pathway and perform a foot screen, backed up by videos and a user guide for the tools. Implementation support was provided by the DON SCN™ project team, which included authors KD and PO. More than 1300 healthcare providers attended DON SCN™ sponsored continuing education conferences provided in 2017 and 2018. In addition, the clinical pathway and associated resources were freely available via the internet and a series of e-learning modules was created. Of 390 individuals who enrolled, 62% (*n* = 242) completed the e-learning courses.

### Outcomes

A customized cross-sectional computer-based survey study was conducted in 2014 and again in 2019, prior to, and following widespread implementation of the diabetes foot care pathway in order to assess its uptake and changes in practice. The data collected compared screening practices, assessment of feet at moderate or high risk for ulceration, and protocols for dealing with urgent cases. The survey items were organized into sections by risk levels (low, moderate, high, urgent) and offered multiple options of care models. Respondents selected options, with more than one option being available for most questions. Respondents could also provide responses to an open-ended question to comment more fully on issues relevant to each risk section (Table 1). The 2014 survey was developed to determine what the current state of foot and wound care was in the province and how currently the different risk levels were being managed. The 2019 survey was a follow-up to determine how the landscape had changed with the implementation of the pathway and HRFT. The 2014 and 2019 surveys were similar with the 2019 version modified to include HRFT as a referral option. In this report the focus was on screening and assessment of individuals with low and moderate risk of foot ulcer.

**Table 1:** Definition of variables

Question number	Variable	Definition / options
**General Questions**		
1	Health zone	Geographic region within Alberta (North, Edmonton, Central, Calgary, South) or other (e.g. Federal)
2	Area of practice	Primary care network, home care, long-term care community (e.g. chronic disease management nurses working in the community), outpatient departments, wound clinics, other
3	Identification of site	Free text (e.g. specific clinic or hospital site)
4	Footcare related continuing education	For all staff in setting within past 2 years; DON SCN sponsored symposia in 2017 or 2018; DON SCN-led workshops in various settings as part of intervention roll-out; Diabetes Canada sponsored events; other
5	Basic foot screening offered	Yes/No
**If Yes to #5, then…**		
6	Screening tool used	AHS screening tool (2019 only), 60-s foot screening tool, other (free text)
7	Screening setting	Free text
8	Risk category seen	Low, moderate, high risk, urgent care required, other
**Questions regarding moderate-risk patients**		
9	Formalized clinic for assessment	Yes (HRFT or other), no
**If Yes to #9, then…**		
10	Management of skin/nail abnormalities	Perform skin/nail care in practice area; provide list of foot/nail care providers in the community; refer to podiatrist or footcare nurse (i.e. private services), refer to HRFT, other (free text), none of the above
11	Management of structural deformities	Provide education regarding self-management; refer to podiatrist or orthopedics; other (free text), none of the above
12	Management of footwear	Provide education/information regarding footwear selection; refer to HRFT; provide referral for footwear/orthotics through AADL; refer for footwear/orthotics without AADL authorization; other (free text), none of the above
13	Management of vascular problems	Perform vascular assessment; refer to HRFT; refer to general practitioner; refer to vascular lab, other (free text), none of the above
14	Vascular assessment tests performed	ABPI; PPG; Pedal pulses; refer to HRFT; other (free text)
15	Management of loss of protective sensation	Treat neuropathic pain (free text); refer to physician/nurse practitioner for treatment of neuropathic pain; refer to HRFT
16	Reassessment frequency	1–3 months; 4–6 months; 7–12 months; no formalized schedule; other (free text)
17	Any other comments about moderate risk patients	Free text
18	Assess high risk/urgent patients	Yes/No. If yes to these, a series of questions was asked. These responses are not reported in this article.

### Data analysis

Data were downloaded from the host server to an Excel spreadsheet by an individual not part of the study team, then anonymized by removing any personal information prior to providing to the study team. Following cleaning to remove duplicate responses (as determined by site-related information, i.e., identical answers to questions 1–3 in Table 1, resulting in *n* = 3 and *n* = 7 responses removed), the categorical data were coded. Qualitative data (comments) were retained verbatim but were not analyzed for this study. Descriptive statistics were compiled. Comparative statistics were performed to assess differences in footcare practices between groups using two-tailed Fisher’s exact test or Chi-square test.

## Results

Total unique responses in 2014 and 2019 were 104 (denominator unknown) and 75 (out of 1005 email invitations, with invitations to multiple personnel at a single site possible), respectively (Table 2). In both years, about 40% (*n* = 44 in 2014 and *n* = 29 in 2019) of responses came from registered nurses. Although the proportion of licensed practical nurses responding increased nearly 5-fold, the overall mix of respondents’ areas of practice was not significantly different between 2014 and 2019 (*p* = 0.051). By site, the respondents represented all five health zones in Alberta although the proportion from Calgary zone increased, while that from North zone decreased (*p* < 0.05). In both sampling years, the most responses came from sites providing primary health care, HC/LTC and outpatient services, totalling 58% (*n* = 100 out of 172 in 2014) and 62% (*n* = 57 out of 91 in 2019) of responses (noting that some sites provided multiple types of services), with similar proportions between years (*p* > 0.05).

**Table 2:** Respondent and site characteristics in 2014 (*n* = 104) and 2019 (*n* = 75)

	2014		2019		*P*-value*
**Profession of Respondent**	N	%	n	%	0.051
Registered Nurse	44	42	29	39	
Licensed Practical Nurse	4	4	14	19	
Manager or Instructor or Educator	22	21	15	20	
Physiotherapist or Occupational Therapist or Pharmacist or Registered Dietitian	8	8	10	13	
Physician or Nurse Practitioner	9	9	5	6	
Other	5	5	2	3	
No response	11	11	0	0	
**Zone**					
North	39	38	16	21	0.022
Edmonton	27	25	20	27	
Central	18	17	13	17	
Calgary	6	6	16	21	
South	13	13	8	11	
Federal or provincial	1	1	2	3	
**Area of practice (more than 1 answer possible)**	**Total 172 responses**	**%**	**Total 91 responses**	**%**	
Primary health care	24	14	21	23	0.27
Outpatient	33	19	13	14	
Acute care	20	12	8	9	
Wound clinic	19	11	7	8	
Homecare or long-term care (HC/LTC)	43	25	23	25	
Community care	19	11	8	9	
Other^a^	11	6	11	12	
No response	3	2	0	0	
**Service level provided**					
Basic foot screening	58	56	55	73	0.016
No	44		19		
No response	2		1		
Assesses for moderate risk	34	33	36	48	0.044
No	62		29		
No response	8		10		
Assesses for high risk	34	33	35	47	0.019
No	60		28		
No response	20		12		

*p-value < 0.05 by Chi-squared Test or Fisher’s Exact Test was considered significant. Analyses did not include “no response” as an option

^a^Other includes categories with < 5 responses: Rural diabetes program, emergency department or intensive care unit, private/independent service, rehabilitation centre, renal clinic, other

**Fig. 1:**
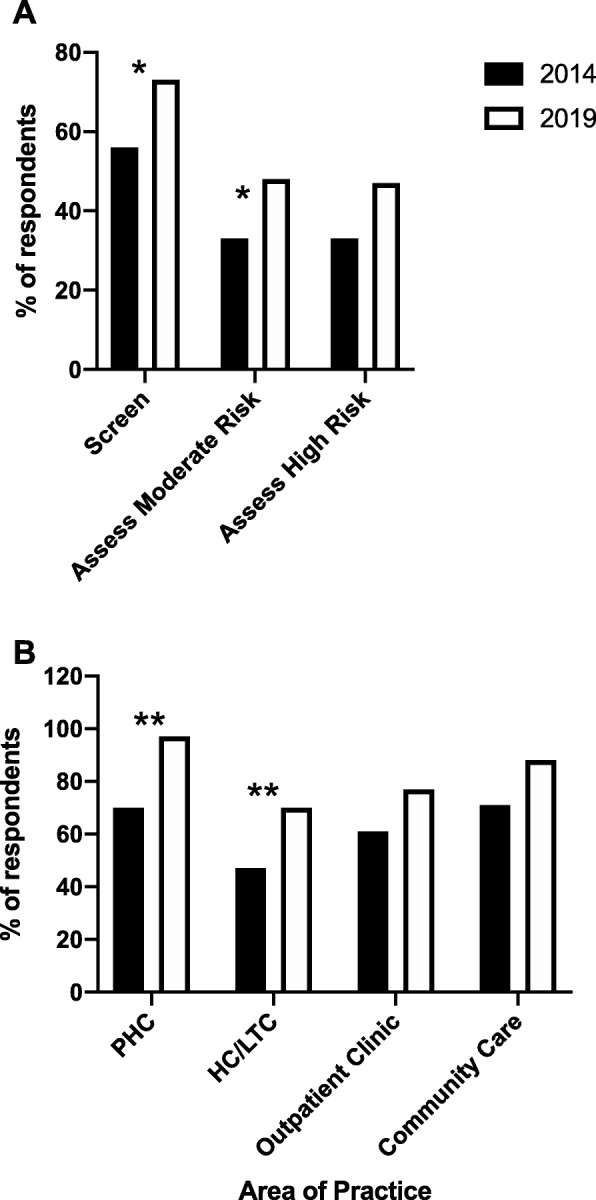
**a** – Proportion of all respondents performing foot screening, or assessing moderate or high risk patients with diabetes. **b** – Proportion of respondents providing primary health care or HC/LTC services that reported providing foot screening for patients with diabetes. *p < 0.05, **p < 0.01 with Fisher’s Exact Test

Figure 1a and Table 2 shows that the proportion of all respondents providing basic foot screening increased from 2014 to 2019 (*p* < 0.05), and this increase was still significant when non-respondents were included in the group not providing screening. An analysis by geographically organized health zones, showed the main increases were in North (*p* < 0.05) and Central (*p* = 0.056) zones, which are primarily rural (Table 3). The main contributors to increased foot screening practices were primary care and HC/LTC sites (Fig. 1b, Table 3), with none of the other areas of practices changing significantly. In 2019 compared with 2014, 5% (*n* = 1) versus 30% (*n* = 6) of primary care sites did not provide basic screening (*p* < 0.01). Similarly 40% (*n* = 7) versus 53% (*n* = 19) of HC/LTC sites did not provide basic screening in 2019 compared with 2014 (*p* < 0.05) (Table 3). In 2019, one-third of respondents had adopted the screening tool developed as part of the DON SCN™ resource toolkit (Table 3).

**Table 3:** Basic Foot Screening – Characteristics of Sites

Provide Basic Screening	2014			2019			*P*-value*
	Yes	No		Yes	No		
**Zone**	N	N	**% Yes**	N	N	**% Yes**	
North	17	21	45	12	3	80	0.031
Edmonton	17	10	63	11	9	55	0.77
Central	8	9	47	11	2	85	0.056
Calgary	4	2	67	13	3	81	0.59
South	11	2	85	6	2	75	0.62
Federal or provincial	1	0	100	2	0	100	ND
Total of all respondents	58	44	57	55	19	74	See Table 2
Non-respondents	2			1			
**Area of practice (more than 1 answer possible)**							
Primary or family practice	14	6	70	20	1	95	0.005
Outpatient	11	7	61	10	3	77	0.452
Acute care	8	11	42	3	4	43	1.00
Wound clinic	8	8	50	7	0	100	0.052
Homecare, long-term care	17	19	47	16	7	60	0.018
Community care	12	5	71	7	1	88	0.623
Other	6	2	75	8	4	67	1.00
No response	1	0	–	0	0	–	
**Screening Tool Used**	N		%	N		%	
**60 s foot screening tool**	50		62	16		31	
**AHS foot screening tool**	–			18		35	
**Other**	31		39	18		35	

*p-value < 0.05 by Fisher’s Exact Test was considered significant, comparing “yes” vs “no responses”. ND, not done

In total, 35% (*n* = 34 out of 96 sites) and 55% (*n* = 36 out of 65 sites) respondents indicated that their site provided assessment of moderate risk patients in 2014 and 2019, respectively (Fig. 1a, Table 2), a significant increase (*p* < 0.05). Of the respondents, the main providers of moderate risk assessment were primary care and HC/LTC, together accounting for 43% (*n* = 18) and 39% (*n* = 22) of the sites providing this service in 2014 and 2019, respectively, which was not significantly different. However, more outpatient (*p* < 0.05) and wound clinics (*p* < 0.01) reported provision of care for moderate risk patients in 2019 than 2014 (Table 4). The service model utilized for moderate risk assessment, in 2014, was most often a hospital or clinic team. In 2019, there were 13 sites reporting utilization of HRFT, which did not exist in 2014.

**Table 4:** Assessment of Moderate Risk – Characteristics of Sites

Have a formalized clinic	2014			2019			*P*-value
	Yes	No		Yes	No		
**Zone**	N	N	**% Yes**	N	N	**% Yes**	
North	11	28	28	5	6	45	0.30
Edmonton	11	12	48	11	7	61	0.53
Central	5	12	29	7	4	64	0.12
Calgary	3	1	75	5	10	33	0.26
South	4	8	33	6	2	75	0.17
Federal or provincial	0	1	0	2	0	100	ND
Total	34	62	35	36	29	55	See Table 2
Non-respondents	8			10			
**Area of practice (more than 1 answer possible)**							
Primary or family practice	10	9	53	13	7	65	0.52
Outpatient	4	14	22	7	4	64	0.048
Acute care	5	13	28	3	3	50	0.362
Wound clinic	4	11	27	6	0	100	0.004
Homecare, long-term care	8	28	22	9	10	47	0.07
Community care	6	10	38	3	4	43	1.00
Other	5	2	71	4	3	57	1.00
**Type of service (more than 1 answer possible)**							
HRFT	–	–		13	–		
Hospital/clinic team	25	–		20	–		
Home care	5	–		4	–		
See referrals	4	–		2	–		
**Services provided (more than one answer possible)**							
**Skin & nail abnormalities**	N [%]			N [%]			
Total number of sites (formalized and unformalized clinics)	96			39			
Skin or nail care provided	44 [46]			21 [54]			0.45
Provide list of foot/nail care providers in the community	33 [34]			26 [67]			0.001
Refer to podiatrist or footcare nurse	33 [34]			29 [74]			< 0.001
Refer to HRFT	–			11 [28]			–
None of the above	8 [8]			1 [3]			–
Other	2 [2]			3 [8]			–
**Structural deformities (bunions, hammertoes)**							
Total number of sites	95 [100]			39			
Provide education regarding self-management	47 [49]			30 [77]			0.004
Refer to podiatrist or orthopedics	63 [66]			31 [79]			0.007
Other	13 [14]			3 [8]			ND
None of the above	12 [13]			0			ND
**Footwear problems**							
Total number of sites / no answer	96			39			
Provide information/education regarding appropriate footwear selection	76 [79]			35 [90]			0.22
Refer to HRFT	–			18 [46]			–
Refer for footwear / orthotics through AADL	50 [52]			20 [51]			1.00
Refer for footwear / orthotics without AADL authorization	26 [27]			14 [36]			0.31
None of the above	7 [7]			1 [3]			ND
Other	20 [21]			4 [10]			ND
**Services provided for vascular problems**							
Total number of sites	96 [100]			39			
Perform vascular (lower limb) assessment	62 [65]			36 [92]			0.001
Refer to HRFT	–			19 [49]			–
Refer to GP for assessment	36 [38]			23 [59]*			0.035
Refer to vascular lab for assessment	24 [25]			15 [38]			0.14
Other	13 [14]			4 [10]			ND
**If vascular assessment performed, procedures used**	Out of 62			Out of 36			
ABPI	44 [71]			16 [44]			0.011
PPG (toe pressures)	37 [60]			17 [47]			0.29
ABPI + PPG (gold standard)	32 [52]			14 [39]			0.29
Pedal pulses	38 [61]			34 [94]			< 0.001
Other	4 [6]			5 [14]			ND
Refer to HRFT	–			17 [47]			–
**Services provided for loss of sensation / neuropathic pain**	Out of 91			Out of 39			
Refer to MD/NP for treatment of neuropathic pain	83 [91]			28 [72]**			0.007
Refer to HRFT	–			18 [46]			–
Address neuropathic pain in their clinic	6 [7]			5 [13]			0.30
Other	2 [2]			1 [3]			ND
None	0			2 [5]			ND
**Frequency of reassessing patients with moderate risk**							
1–3 months	15 [16]			8 [21]			0.62
4–6 months	11 [12]			11 [28]			0.039
7–12 months	8 [8]			3 [8]			1.00
No formalized schedule	48 [53]			7 [18]			< 0.001
Other	7 [8]			9 [23]			ND

Statistical analysis used Fisher’s Exact Test

Abbreviations: *ABPI* Ankle-brachial pressure index, *AADL* Alberta Aids to Daily Living, *GP* General practitioner, *HRFT* High risk foot team, *MD* Medical doctor, *NP* Nurse practitioner, *PPG* Photoplethysmography toe pressure, *ND* Not done

**Fig. 2:**
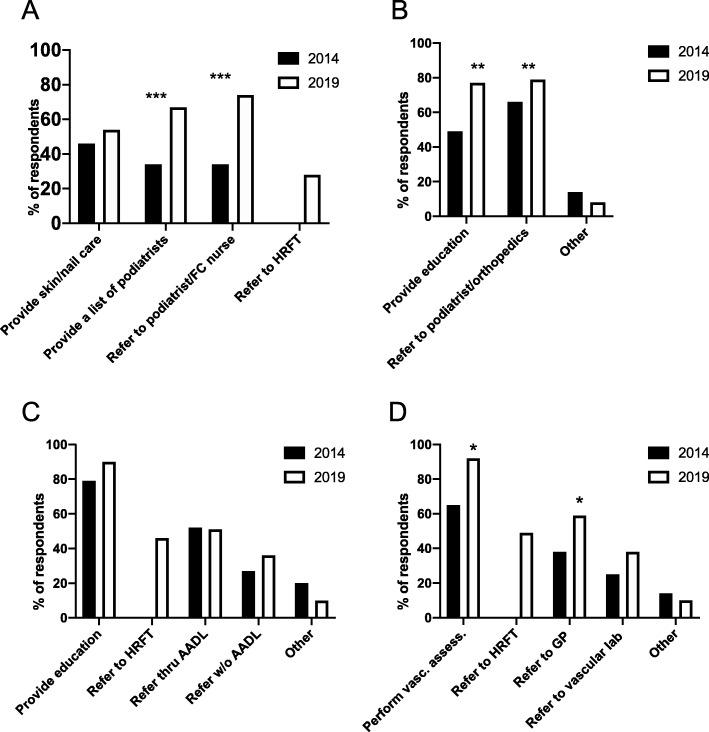
Provision of service to patients with moderate risk feet. **a** – Nail and skin care services provided. **b** – Services for structural deformities provided. **c** – Services for specialty footwear provided. **d** – Performance of vascular assessment and related referrals for peripheral artery disease. Comparing 2014 to 2019, *p < 0.05, **p < 0.01, *** *p* < 0.001 by Fisher’s exact test. Data were analysed using raw counts and transformed to % respondents for presentation

With regard to the services provided, between 2014 and 2019, there were significant increases in the proportion of sites providing patients with lists of foot/nail care providers in the community (*p* < 0.001) and sites providing referrals to podiatrists or footcare nurses (*p* < 0.001) (Fig. 2a, Table 4). Similarly, for practices related to structural deformities, an increased proportion of sites provided relevant education (*p* < 0.01) and provided referrals to podiatrists or orthopedic specialists (*p* < 0.01) (Fig. 2b, Table 4). However, no differences in education about footwear or referral to providers of specialty footwear were detected (Fig. 2c), with the exception that referral to HRFT was a new option available in 2019. In 2019 a significantly (p < 0.01) bigger proportion of respondents reported that their sites performed assessment of vascular problems than in 2014 (Fig. 2d, Table 4). These included referral to general practitioners (*p* < 0.05) along with HRFT and vascular laboratories. Finally, services provided for loss of sensation and neuropathic pain were found to be similar between service models, with referral to a physician or nurse practitioner predominating but HRFT referred to by nearly half of the sites in 2019 (Table 4). Frequency of reassessing patients with moderate risk changed, with a smaller proportion of clinics not having a formalized schedule (*p* < 0.001) and more clinics reassessing at 4–6 months in 2019 vs 2014 (*p* < 0.05) (Table 4).

We compared practices in assessing and caring for moderate risk patients reported by sites utilizing specified HRFT (*n* = 13) compared with those utilizing other models of care delivery (other types of clinical teams) (*n* = 20) using 2019 data. The areas of practice represented in the two care models were not significantly different (*p* > 0.05) (Table 5). The sites reporting HRFT were located more in urban areas than the clinic/hospital teams (*p* < 0.05). More clinic/hospital teams than HRFT provided skin and nail care (75% versus 63%, (*n* = 15 versus *n* = 5, *p* < 0.1). Referral to podiatrists and assessment of structural deformities was similar between care models. HRFT were more likely to hold AADL authorization than other sites (*p* < 0.1), meaning that patients could be referred and have a portion of costs covered by Alberta’s publically-funded insurance plan. With regard to assessment of vascular problems, all sites provided this service but clinic/hospital teams were more likely to refer patients to their general practitioner (*p* < 0.05). HRFT were more likely to assess pedal pulses than clinic/hospital teams (*p* < 0.1) but no differences were detected in use of other diagnostic modalities. Finally, with regard to assessing loss of protective sensation, practices were similar between the care models (Table 5).

**Table 5:** Comparison between sites utilizing HRFT vs other care models for assessing moderate risk patients

	All	HRFT	Clinic/ hospital team	*P*-value
N unique sites	36	13	20	
PHC	10	5	5	0.574
Outpatient	6	3	3	
Acute care	1	0	1	
Wound clinic	4	2	2	
HC/LTC	9	5	4	
Community care	1	0	1	
Private/independent	2	0	2	
Multiple areas of practice	6	1	5	
N for geographical location:				0.015
Metro & Urban	21	11	8	
Rural	10	2	7	
Remote	2	0	2	
First Nations	3	0	3	
**Services provided**		N (%)	N (%)	
Skin and nail care	Provide skin & nail care	5 (38)	15 (75)	0.067
	Provide a list of community resources	11 (85)	13 (65)	0.264
	Refer to podiatrist	9 (69)	18 (90)	0.184
Assess structural deformities	Provide education	11 (85)	17 (85)	1.00
	Refer podiatrist or orthopedic specialist	11 (85)	18 (90)	
	Other	0	2 (10)	–
Address footwear problems	Provide education	13 (100)	19 (95)	0.501
	Refer to AADL	9 (69)	7 (35)	0.0799
	Refer without AADL authorization	8 (62)	8 (40)	0.296
	Other	5 (38)	5 (25)	
Assess vascular problems	Perform vascular assessment	13 (100)	20 (100)	1.00
	Refer to GP	5 (38)	16 (80)	0.0265
	Refer to vascular lab	10 (77)	10 (50)	0.1595
Vascular assessment methodology	ABPI	7 (54)	8 (40)	0.4928
	PPG	9 (69)	8 (40)	0.151
	ABPI + PPG	7 (54)	7 (35)	0.472
	Pedal pulses	12 (92)	12 (60)	0.0560
	Perform all 3 tests	7 (54)	7 (35)	0.472
	Other	1 (8)	1 (5)	
Assess loss of protective sensation	Refer to physician	10 (77)	18 (90)	
	Treat neuropathic pain	3 (23)	2 (10)	

Statistical analysis using Fisher’s Exact Test. For discussion purposes, *p* < 0.1 was considered significant given the small number of sites available for comparison

Abbreviations: *AADL* Alberta Aids to Daily Living, *ABPI* Ankle-brachial pressure index, *GP* General practitioner, *HC/LTC* Homecare/long-term care, *PHC* Primary health care, *PPG* Photoplethysmography toe pressure

Metro, urban, rural and remote were defined according to Alberta Health Services and Alberta Health criteria [28]. *N* = 3 sites responded “other” (one referred to home care, two were themselves referral sites)

## Discussion

Diabetic foot diseases compromise the quality of life of people with diabetes [29] and are costly to the health system [1, 30]. Yet in Canada, primary prevention practices such as screening are performed less frequently than for other co-morbidities of diabetes such as hypertension [16, 31]. Comprehensive foot care practices including screening and measures to prevent ulcer development are recommended, with multidisciplinary teams involved in patient care for people with diabetes. Multidisciplinary teams such as foot protection services or HRFT have been deployed in a number of jurisdictions to increase screening and risk stratification [32], access to specialized care [10, 11] and reduce outcomes such as severe infection and lower limb amputations [32, 33]. In Alberta, Canada foot protection services, named HRFT, supported by the DON SCN™ were first constituted in 2015 at three pilot sites and have since spread with a total of six operational sites in the province. A clinical care pathway, training in foot screening, referral guidance and additional resources were provided. The combination of these activities was shown to increase screening activity, particularly in primary health care and HC/LTC settings along with increased proportion of reporting sites that provide services for the assessment of moderate-risk patients.

Both clinical and cost-effectiveness are essential to sustaining innovations in healthcare delivery. Interventions targeting healthcare organizations to improve secondary prevention of DFU are effective in reducing ulcer recurrence and lower limb amputations [21, 33]. Moreover, timely access to wound care specialists results in less severe presentation and faster healing than when access to specialists is delayed [8, 34]. Preliminary exploration of lower limb amputation rates in Alberta after 1-year follow-up indicated a small reduction of 0.5% and a significant net monetary benefit of $3000 per patient-year, consisting of $3500 health utilization cost avoidance versus $500 intervention cost [35]. When foot protection services were implemented in a hospital in Ireland they were proven to reduce DFU and be cost-effective [36]. Likewise, a Scottish analysis found a 0.3% reduction in lower limb amputation after introducing a national strategy for screening and risk stratification [32].

From our survey, which components of the clinical pathway, resource toolkit and training were found most valuable in facilitating organizational change could not easily be identified. However, a separate survey conducted by the DON SCN™ only in primary care settings (for program evaluation purposes) found that the most used resources were the foot screening tool, followed by the Diabetes Foot Risk Assessment Triage Referral form and its associated process guidelines, and the patient resource. These materials were used by more than 30% of the respondents. E-learning modules and the Provider’s Guide for the pathway were used by less than 25% of respondents (K. Dmytruk and M. Mainville, personal communication). Annual screening is recommended in Canada and internationally [26, 37]. A review of the utility of screening in primary care of all people with diabetes yielded only weak evidence for benefit [38] but in the context of a comprehensive care plan, is the first step to identifying risk and ensuring that people with diabetes can access specialized care expeditiously [26]. Our survey found an overall increase in screening from 57 to 74% of respondents predominantly in primary health care and HC/LTC sites, which is important, because foot problems identified early increases the potential to avoid overt ulceration [39]. Uptake of the AHS foot screening tool was strong in 2019, likely due to the training provided in its use. Unfortunately, actual screening rates on individual patients in primary health care and HC/LTC are not easily tracked. However, based on the data presented here, we predict that individuals self-reporting an annual foot screen would increase from the 40% recorded in a cohort prior to implementation of the diabetes foot care pathway [16]. To further increase uptake of diabetes foot screening into clinical practice, embedding the screening tool into the electronic medical records would allow for automated reminders and tracking of foot screens performed.

An online patient education resource and handouts for each risk level were developed by the DON SCN™. Patient education is recommended [26] even though evidence for effectiveness is conflicting [40, 41]. In particular, a single education session focused only on increasing knowledge is unlikely to provide lasting behaviour change [41]. Individuals with diabetic foot disease express complex emotional and behavioral responses to their condition and may feel they lack control over their ability to prevent re-ulceration [42], thus the provision of simple leaflets may be insufficient support.

When screened patients present with skin, nail, anatomical or sensory abnormalities but not skin breakdown or ulceration, they are defined as moderate-risk in the AHS-developed screening tool and the pathway recommends footcare education and referral to a footcare nurse or podiatrist by the family physician or HRFT [23]. In 2019, about half of respondents referred such patients to family physicians, the other half to HRFT (with some overlap). Improvement in practices for moderate-risk patients in 2019 included more sites following the recommended [23] 4–6 months follow-up increasing 1.7-fold and an increased proportion of sites performing vascular assessment, which requires performing specific diagnostic tests by 1.3-fold compared with 2014. Training and video modules of assessment were provided to facilitate uptake. Patients with peripheral artery disease have 2-fold higher risk of major lower limb amputation [43] so the increased proportion of sites performing vascular assessment is encouraging because a systematic review found that timely referral of patients with peripheral artery disease can reduce morbidity and mortality [44]. Access to HRFT following community-level screening is important, in particular forming networks with a HRFT hub [32]. It was facilitated in the clinical care pathway by the Triage Referral Form provided to those in primary care performing screening.

We identified differences in clinical practices between clinic/hospital teams and HRFT, which received specific training in the pathway and resource toolkit use. Specifically, clinic/hospital teams were more likely to provide skin and nail care than HRFT, which was associated with their service to mainly remote and First Nations communities. This might reflect lack of community services (e.g., podiatry) for such care. Indeed, inspection of the sites providing skin/nail care by the clinic/hospitals revealed that 12 out of 12 rural/remote/First Nations sites provided this service versus only 3 out of 8 urban sites. Provision of such services in rural settings is important, perhaps even more so in First Nations communities because the prevalence of neuropathy is high and patients tend to be younger than in non-First Nations populations [45, 46]. Referral of patients to family doctors versus a specialized vascular laboratory was noted for the clinic/hospital teams, possibly also related to access variation imposed by geography. Referral practices for therapeutic footwear did not change but this may be more related to restrictions on prescribing and insurance coverage than lack of attention to pathway recommendations. Because properly-fitted footwear is important for preventing ulcers [47], development of policies and clear criteria for more universal provision of therapeutic or custom footwear and offloading inserts would be beneficial.

The strengths of this study include attempts to reach a broad base of respondents from all settings where diabetic foot care is performed, and ability to compare with data collected prior to implementation of the diabetic foot care pathway. However, we acknowledge some weaknesses. The customized survey was not validated and selection bias is probable because sites with a strong interest or expertise in diabetic foot care were more likely to respond than those with less investment in that area. Moreover, the professions of non-respondents were unable to be fully documented because of the untargeted recruitment, particularly in 2014. Also, sites receiving multiple invitations to participate may have designated the most knowledgeable member of the team to respond to the survey to avoid duplication of effort. Nurses may have been over-represented (about 40% of respondents), however, nurses play an important role in foot screening and foot care and thus may be the most knowledgeable of their site’s practices. Although we were unable to directly compare sites’ responses in 2019 versus 2014, we could document trends in improved diabetic foot care practices in Alberta. Ability to assess the benefits of HRFT was limited by the small number of respondents and differences in geography between HRFT and the comparator group of hospital/clinic teams. Finally, this analysis provides an overview of the uptake of the comprehensive diabetes foot care clinical pathway and resource toolkit but further work should include validation of the foot screening tool and evaluation of the effectiveness of the patient education resources to improve self-care.

In conclusion, this study supports that a multi-faceted, concerted approach to improve diabetic foot care improves awareness of healthcare providers and uptake of appropriate screening in primary care and HC/LTC settings in Alberta, Canada. Provincial outreach and training provided by the DON SCN™ facilitated uptake of the clinical pathway. Preliminary data are consistent with cost avoidance and reduction in foot-related complications in persons with diabetes and are consistent with reports from other jurisdictions. HRFT provide increased expertise for assessment and procedures for patients with moderate risk of ulceration so that individuals screened in primary healthcare, homecare or other settings can be referred. This comprehensive model has the potential to reduce progression of foot problems and overall health services utilization.

## Supplementary information

**Additional file 1.**

## Data Availability

Diabetic foot care clinical pathway resources and information are available as Supplemental Materials to this article and at https://www.albertahealthservices.ca/scns/Page10321.aspx. The datasets used and/or analysed during the current study are available from the corresponding author on reasonable request.

## Abbreviations

ABPI: Ankle-brachial pressure index
AADL: Alberta Aids to Daily Living
AHS: Alberta Health Services
DFU: Diabetic foot ulcer
DON SCN™: Diabetes, Obesity and Nutrition Strategic Clinical Network™
GP: General practitioner
HC/LTC: Homecare/long-term care
HRFT: High risk foot team
MD: Medical doctor
NP: Nurse practitioner
PPG: Photoplesthysmography toe pressure
RNAO: Registered Nurses Association of Ontario
