# Supplementary material for: Organizational changes in diabetic foot care practices for patients at low and moderate risk after implementing a comprehensive foot care program in Alberta, Canada
Source: J Foot Ankle Res. 2020 May 19;13:26. doi: 10.1186/s13047-020-00393-0 (PMC7236492; doi:10.1186/s13047-020-00393-0)
Supplement: Supplementary file 1 — Additional file 1. [file 13047_2020_393_MOESM1_ESM.pdf]

Alberta Health Services

Diabetes Foot Care Clinical Pathway
Healthcare Provider's Guide

Diabetes, Obesity & Nutrition Strategic Clinical Network™

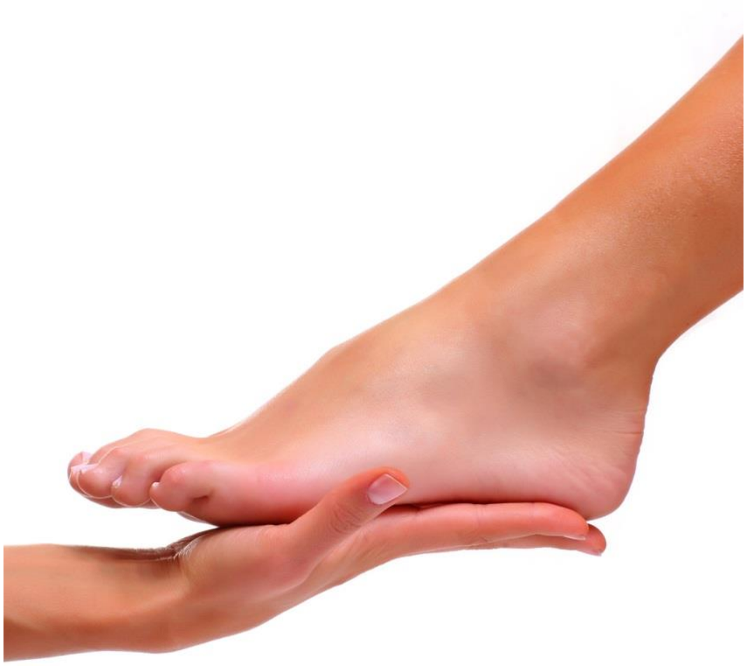

Version 2.0
Last updated June 26, 2019

Acknowledgement
This healthcare provider's guide has been adapted from the New Brunswick Diabetes Foot Care Clinical Pathway

©2018 Alberta Health Services, Diabetes, Obesity & Nutrition Strategic Clinical Pathway.

This work is licensed under a Creative Commons Attribution-Non-commercial-Share Alike 4.0 International license. You are free to copy, distribute and adapt the work for non-commercial purposes, as long as you attribute the work to Alberta Health Services and abide by the other license terms. If you alter, transform, or build upon this work, you may distribute the resulting work only under the same, similar, or compatible license. The license does not apply to content for which the Alberta Health Services is not the copyright owner.

This material is intended for general information only and is provided on an "as is," "where is" basis. Although reasonable efforts were made to confirm the accuracy of the information, Alberta Health Services does not make any representation or warranty, express, implied or statutory, as to the accuracy, reliability, completeness, applicability or fitness for a particular purpose of such information.

Table of Contents

Introduction 1
The Diabetic Foot Care Clinical Pathway Resources 1
Overview of the Diabetes Foot Screening Tool 1
The Foot Screening Process 2
Foot Screening Tool 3
Performing the Foot Screening Exam 4
Skin Assessment 4
Nail Assessment 7
Structure/Anatomy Assessment 8
Sensation Testing 10
Vascular Assessment – Identify PAD (Peripheral Arterial Disease) 13
Footwear Assessment 16
Proper Foot Care 17

Introduction

People with diabetes should be assessed for their risk of developing a diabetic foot ulcer when they are first diagnosed with diabetes and at least once a year thereafter. Patients at higher risk should be assessed more frequently. Completing a foot screen, coupled with ongoing re-assessments and timely appropriate interventions can greatly improve the patient's overall quality of life.

Early detection of risk factors and ongoing patient follow-up has been found to reduce the development of foot ulceration and subsequently reduce amputations. The majority of amputations related to diabetes are preceded by a diabetic foot ulcer; of which, up to 85% are preventable.

The Diabetes Foot Care Clinical Pathway (DFCCP) has been developed to enhance early detection and timely treatment of diabetes related foot problems. The DFCCP resources are intended to support healthcare providers in performing diabetes foot screening exams, using the Diabetes Foot Screening Tool and referring patients to the most appropriate healthcare provider(s) within the recommended time frames.

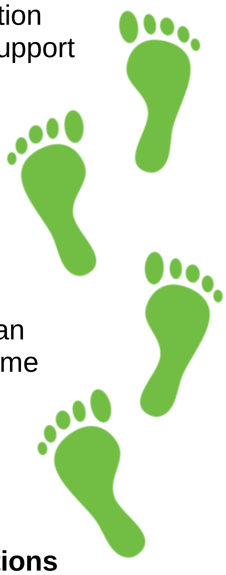

The Diabetic Foot Care Clinical Pathway Resources

- **Diabetes Foot Screening Tool** – Guides you in assessing the foot, identifying risks of ulceration and classifying the level of patient risk
- **Diabetes Foot Risk Assessment Triage Referral Form** – Available for communities that can refer to a High Risk Foot Team; helps direct the patient to the correct service in a specified time frame contingent on the assessed level of risk
- **Diabetes Foot Care Clinical Pathway Healthcare Provider's Guide** (this resource) – Provides supplemental information to assist the assessor in completing the foot screening sections and identify level of risk
- **Referral Process Guideline** - High level overview of the referral and follow-up guidelines
- **Foot Care for People with Diabetes: Low, Moderate and High Risk Care Recommendations Booklet** – A patient education booklet which describes the various risk levels, how to take care of their feet and prevent progressing to a higher level of risk
- **Low, Moderate and High Risk Diabetic Foot Patient Information Sheets** – A condensed, single-page, patient handout based on each respective level of risk

Overview of the Diabetes Foot Screening Tool

The Diabetes Foot Screening Tool is designed to help you thoroughly examine a patient's foot and determine the level of risk. There are 6 screening components and 4 levels of risks: low, moderate, high, and urgent. The form also includes an area to record your findings for each foot (right and left).

- Low risk findings indicate a normal foot assessment with no significant skin, nail, anatomical, vascular or sensory abnormalities and require a foot assessment annually
- Moderate risk findings include skin, nail, anatomical or sensory abnormality with no skin breakdown/ulcer and/or inadequate footwear. Patients with moderate risk findings need to be addressed within one month of assessment and follow-up every 4 to 6 months
- High risk findings are characterized by skin breakdown/ulcer, and/or impaired circulation with no signs of infection or cellulitis. Patient's with any high risk finding need care within 2 weeks of assessment and follow-up every 1 to 4 weeks
- Urgent findings such as cellulitis, draining ulcer, acute Charcot joint collapse, gangrene, cold white painful foot or part thereof require immediate assessment and treatment (within 24 hours)

The Foot Screening Process

There are several steps in the Diabetes Foot Care Clinical Pathway:

- Examination and assessment of the patient’s feet
    - Identify state of skin and nails, deformities, arterial compromise, and neuropathy
    - Identify state of their shoes (inside and out)
- Complete the two pathway forms (screening and triage)
- Make appropriate and timely referrals
- Provide patient education and follow-up

1. Have the patient remove their shoes. It is important to assess both feet and footwear
2. Complete the ***Diabetes Foot Screening Tool*. **Assess and record your findings for each foot
3. Determine the patient’s ***Level of Risk*** (the overall risk is determined by the highest level assessed for either foot)
4. Identify what referrals are needed to address the patient’s assessed needs and complete the ***Diabetes Foot Risk Assessment Triage Referral Form***
5. Refer the patient to appropriate service(s)
6. If referring to a High Risk Foot Team (HRFT), include a copy of both DFCCP forms with your referral (Diabetes Foot Screening Tool & Diabetes Foot Risk Assessment Triage Referral Form)
7. Provide the patient with education, self-management strategies and resource handout(s) **“Foot Care for People with Diabetes: Low, Moderate and High Risk Care Recommendations”**
8. Provide the patient with a follow-up appointment

Note:

You can go to https://www.albertahealthservices.ca/scns/Page13331.aspx to identify if there is a High Risk Foot Team in your area. If your area does not have a HRFT, refer your patient to the most appropriate local healthcare provider to address the patient’s identified needs (e.g. podiatry, foot care nurse, vascular surgeon, infectious disease, dermatology, etc.)

Foot Screening Tool

Alberta Health Services

Patient Label

Diabetes Foot Screening Tool

EXAM	FINDINGS	R	L	RISK
SKIN	Normal intact skin – healthy or dry *check in between toes			LOW
	Callus/Corn/Fissure/Crack not bleeding or draining			MODERATE
	Prior history of Diabetic Foot Ulcer(s) ulcer in remission			
	Blister = B or Hemorrhagic callus = HC			HIGH
	Fissure or Crack Bleeding or draining = F			
	Diabetic Foot Ulcer – Not infected and/or with intact dry black eschar = U			
	**Infected Diabetic Foot Ulcer or wet gangrene**			*URGENT*
NAILS	Normal well-kept with minimal discoloration			LOW
	Missing, sharp, unkept, thickened, long or deformed			MODERATE
	Infected ingrown nail			
STRUCTURE ANATOMY	Normal no noted visual abnormalities			LOW
	Decreased range of motion at ankle or toe joint			MODERATE
	Deformities Bunion/Hammer or claw toes/overlapping toes			
	Structure Fallen Arch/ Rocker bottom foot/stable Charcot foot			
	Previous amputation **X** over location or draw/describe on diagram			
	Redness over any structural deformities pressure related			HIGH
	**Red, hot painful joint or acute Charcot foot**			*URGENT*
SENSATION Testing for LOPS	Normal sensation using 10 g monofilament at the 5 predetermined sites			LOW
	Sensation of numbness/tingling/throbbing/burning			MODERATE
	Absent or altered sensation at one or more of the five sites			
	**Acute onset of pain in a previously insensate foot**			*URGENT*
VASCULAR Testing for Arterial Compromise	Normal pulses normal capillary refill			LOW
	Signs of Ischemia (PAD)<br>Cool skin with pallor, cyanosis or mottling, and/or dependent rubor			HIGH
	One or more pulses not palpable or audible (Doppler)			
	**Absent pedal pulses with cold white painful foot or toes**			*URGENT*
FOOTWEAR	Appropriate accommodates foot shape			LOW
	Inadequate Footwear			MODERATE
	Inappropriate Footwear causing pressure/skin breakdown			HIGH

SAMPLE

Instructions: *Refer to Health Provider's Guide to Diabetes Foot Screening*

Mark ulceration location (U). Mark other areas of specific concern: blister (B), draining fissure/crack (F), hemorrhagic callus (HC), and previous amputation (X).

Sensation Testing (monofilament)

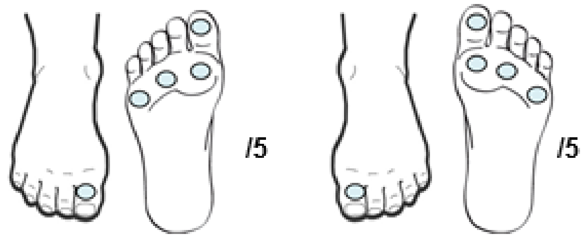

RIGHT LEFT

Fill in if no sensation ●

Leave blank if sensation present ○

Identify any wounds and location on the foot or toe(s)

Date: ____________________ Signature: ____________________

Primary Care Site ____________________

Comments: ____________________

Adapted from New Brunswick Diabetes Foot Care Clinical Pathway

Performing the Foot Screening Exam

Skin Assessment

Findings	Risk & Action Plan	Screening Tips
**Normal skin**<br>(intact and healthy)<br>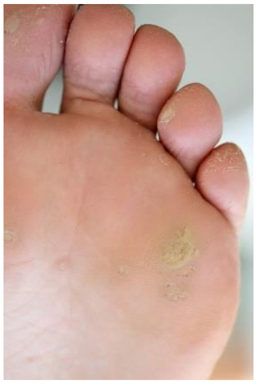	**LOW RISK**<br>• Normal intact skin or,<br>• Skin may be dry or too moist<br>**Intervention**<br>• Dry skin - requires a moisturizer<br>• Excessive moisture between toes (maceration) - may require a wicking or drying agent<br>**Education**<br>• Provide low risk diabetes foot information<br>**Follow-up**<br>• Foot exam/screen required annually	• Inspect top & bottom of ***both*** feet<br>• Check ***in-between toes*** for skin breakdown or excess moisture<br>• Check skin temperature (run back of your hand down front of shin from knee to toes); ***compare*** both feet;<br>↑ temperature in one limb is often the first sign of inflammation/infection<br>• Moisturizers should be medical grade<br>• Consider recommending patient purchase Diabetic Socks
**Callus or corn**<br>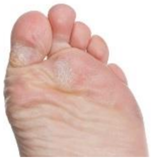<br>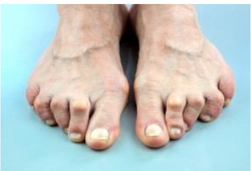<br>**Fissure or crack**<br>(no bleeding or draining)<br>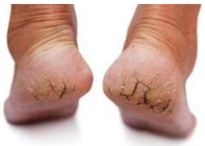<br>**Fungus**<br>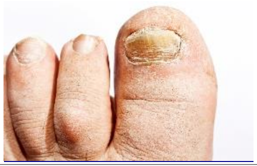<br>**Prior history of foot ulcer**<br>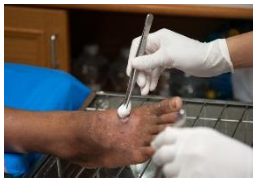	**MODERATE RISK**<br>**With or without LOPS**<br>If assessment indicates only LOPS and no other findings the Primary Provider can address the identified patients risk factors<br>With LOPS **<u>PLUS</u>** any of the following, a referral to the HRFT may be warranted<br>**Assessment**<br>• Assess footwear<br>• Look for and identify any pressure areas, redness, bruising, abrasions or cuts<br>• Identify corns, calluses, cracks or fissures<br>• Identify any dermatological concerns (e.g. yeast/fungus)<br>• Ascertain if any past ulcerations have occurred (ulcer in remission)<br>**Intervention**<br>• Fissures require topical treatment to support closure<br>• Treat any skin conditions/open areas<br>• Refer to an AADL authorizer for therapeutic footwear if required<br>**Education**<br>• Provide moderate risk diabetes foot information<br>**Follow-up every 4-6 months**	• Loss of Protective Sensation (LOPS) is the leading predictor of foot ulceration. It is a crucial turning point for the patient in regards to their risk and need for additional interventions such as<br>○ Professional nail care<br>○ Corns and calluses should be managed by a medically trained foot care provider *<br>○ Appropriate offloading footwear<br>○ An increased need for monitoring and follow-up<br>***Foot care providers**: podiatrist, foot care nurse or other medically trained professional competent in providing skin & nail care. These services may have a fee and a referral is likely required<br>• Footwear is the #1 cause of foot trauma and contributes to skin difficulties<br>• Alberta Aids to Daily Living (AADL) authorizers are part of HRFT's<br>• Re-ulceration is a lifelong risk; the tensile strength of damaged tissue is only 80% of its former strength

Skin Assessment continued

<table>
<tr><th>Findings</th><th>Risk & Action Plan</th><th>Screening Tips</th></tr>
<tr>
<td>Blister
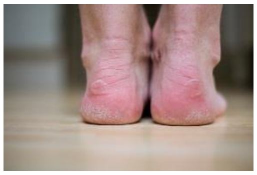</td>
<td rowspan="5">HIGH RISK
Any high risk findings should be referred to the HRFT or local specialist with an appointment to be seen within 1-2 weeks
Assess for
• Blister(s)
• Hemorrhagic callus
• Bleeding draining fissure or crack
• Diabetic foot ulcer
• Redness over structural deformity
• Signs of arterial insufficiency
• One or more pedal pulses not palpable or audible
• Footwear causing pressure or skin breakdown
Intervention
• Diabetic Foot Ulcer
  o Initiate appropriate wound dressing protocol until patient can be seen by the HRFT
  o Treat infection
  o Consider offloading affected foot
Education
• Provide high risk diabetes foot information
Follow-up
• Foot assessment every 1-4 weeks</td>
<td rowspan="5">Signs of inflammation or infection include:
• ↑ skin temperature
• Swelling
• Redness
• Increased exudate
• Odor
• Pain where there was no pain before
• Unexplained increase in blood glucose
• Increase in wound size
*Antibiotic Therapy: Guided by Diabetic Foot Infection Guidelines in BUGS & DRUGS or alternate resource

A dry black eschar should be left intact if eschar is not boggy, no exudate, and no pain or redness

Any debridement of eschar should only be completed after a lower leg assessment has been completed by a trained health care professional – requires a referral to most appropriate health care provider

Walking on thick calluses can damage the healthy skin underneath. This may lead to an accumulation of blood or an ulcer beneath the callus

In a neuropathic foot, a callus is 11 times more likely to ulcerate than a site without a callus</td>
</tr>
<tr><td>Hemorrhagic callus (bleeding beneath)
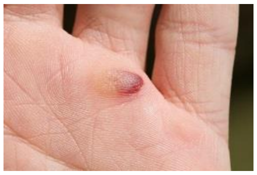 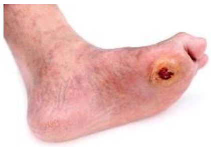</td></tr>
<tr><td>Fissure or crack (bleeding or draining)
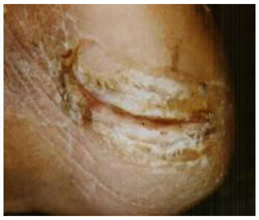</td></tr>
<tr><td>Non infected ulcers/
Dry black eschar
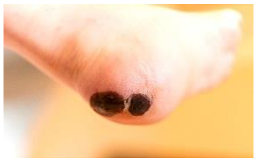</td></tr>
<tr><td>Mild/superficial wound infection NOT requiring hospital admission
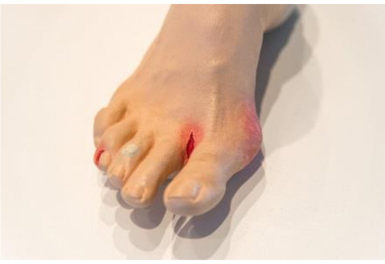</td></tr>
</table>

Skin Assessment continued

Findings	Risk & Action Plan	Screening Tips
**Infected, draining ulcer**<br>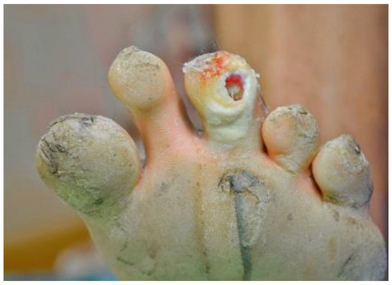<br>**Red, hot, swollen foot or cellulitis**<br>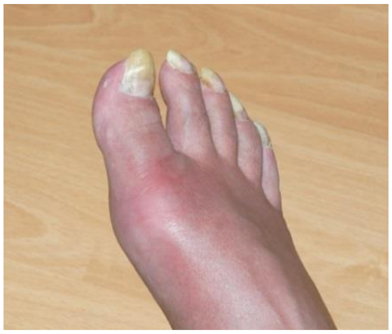	**URGENT RISK**<br>**Triage for immediate treatment if any of the following are present**<br>• Infection - draining diabetic foot ulcer or wet gangrene<br>• Red, hot, swollen foot<br>• Acute Charcot foot<br>• Acute pain in a previously insensate foot<br>• Absent pedal pulses with cold, white, painful foot or toes<br>**Intervention**<br>• Antibiotic therapy required guided by Diabetic Foot Infection Guidelines in BUGS & DRUGS or alternate resource<br>• Consult Infectious Disease (ID) if appropriate<br>• Total offloading/non-weight bearing of foot (Charcot, ulcer)<br>• May require hospital admission<br>• Requires close medical monitoring	• These situations often require a hospital admission<br>• Wet gangrene or non-intact eschar presents as necrosis of tissue with excessive moisture; often indicates infection<br>• In the presence of advancing cellulitis, consider sepsis<br>• Osteomyelitis is assumed (89%) in any person with diabetes whose wound probes to bone. This situation requires IV antibiotic intervention<br>• Offloading the foot decreases the risk of further trauma<br>• Neuropathy can impact a patient's ability to feel pain. When pain occurs in an insensate foot this is an indicator of an urgent situation<br>• Pain often accompanies these conditions and should be addressed at time of visit<br>• A referral to the High Risk Foot Team is recommended once the patient situation has stabilized

Helpful education hints for skin care

- The skin is the first barrier to infection and any skin breakdown can lead to limb-threatening consequences for people with diabetes
- Encourage patients to wash their feet daily and to make sure that they dry between toes
  - Do not moisturize in-between toes
  - Do not soak feet as part of foot care regime
- Review patient handouts to highlight recommendations for patient self-care and management

Treating foot ulcers can be complex and requires the care of several disciplines and sites of service to manage:

- Glycemic control
- Pressure relief or offloading (therapeutic footwear)
- Infection control
- Identify lower extremity vascular status – completing a lower limb assessment which includes ABPI (ankle brachial pressure index) **and** PPG (photoplethysmography) toe pressures
- Local wound care – best achieved with an interdisciplinary approach from professionals with expertise in chronic ulcer care

__Offloading__ is used to re-distribute plantar pressure to the foot. Most foot ulcers occur at areas of increased pressure. To heal an ulcer, this pressure must be minimized or avoided.
Several devices can be used for offloading. __The HRFT or Specialty Wound Care Team__ can assess the patient's plantar pressures and recommend the best offloading approach (e.g. crutches for non-weight bearing, Darco shoes, custom orthotics, removable cast boots (AFO) or total contact casting).
These devices are to be worn whenever the patient is on their feet. Compliance is often an issue therefore patient understanding is key to encouraging adherence.

Nail Assessment

Findings	Risk & Action Plan	Screening Tips
**Normal nails, well-kept minimal discoloration**<br>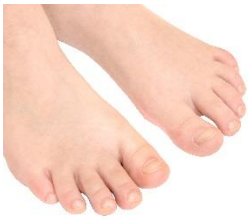	**LOW RISK**<br>**Education**<br>• Provide low risk diabetes foot information<br>**Follow-up**<br>• Foot exam/screen required annually	• Inspect the toenails<br>• Support self-care strategies through education<br>• Increased patient understanding of the importance of proper nail care has shown to improve adherence and reduces risk factors
**Missing, sharp, unkept, thickened, long or deformed**<br>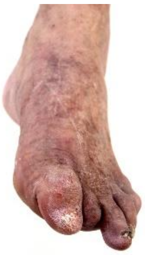<br>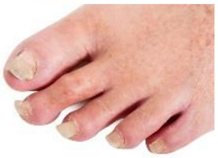<br>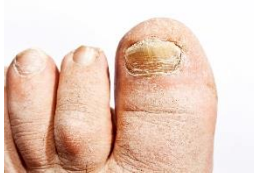	**MODERATE RISK**<br>**Assessment**<br>• Check if sharp or unkept nails are causing cuts or wounds<br>**Intervention**<br>• Refer patient for nail care by a professional foot care provider*<br>• Patient should be seen within 1 month of assessment<br>**Education**<br>• Provide moderate risk diabetes foot information<br>**Follow Up**<br>• Foot assessment **every 4-6 months**	• Thickened nails may indicate vascular or fungal infections<br>• Patients with difficulty reaching their toes and/or with sensory abnormalities may need assistance with nail care<br>• Nail care can be performed by a competent family member. If no family member is available or is difficult, then nail care should be done by a trained foot care provider*<br>***Foot care providers**: podiatrist, foot care nurse or other medically trained providers competent in providing skin and nail care to people with diabetes. These services may have a fee and a referral is likely required<br>• Routine skin & nail care **is** not often provided by the High Risk Foot Team
**Infected Ingrown toenail**<br>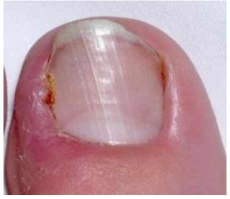<br>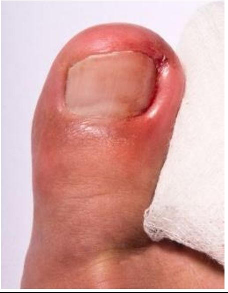	**Assessment**<br>• Identify type of infection<br>**Intervention**<br>• Treat infection<br>• Refer for advanced intervention if deemed warranted (e.g. removal of nail or advanced nail care)<br>**Education**<br>• Provide moderate risk diabetes foot information<br>**Follow-up**<br>• Foot assessment **every 4-6 months**	• Ingrown nails can quickly lead to serious foot complications<br>• Systemic antibiotics should be initiated prior to referral if indicated<br>• Recommend antibiotic therapy guided by Diabetic Foot Infection Guidelines in BUGS & DRUGS or alternate resource. Consider Infectious Disease (ID) consult<br>• Referrals may include:<br>○ Surgical intervention for removal of deformed or ingrown nail<br>○ Dermatology<br>○ Podiatry<br>○ Foot Care Nurse

Structure/Anatomy Assessment

<table>
<tr><th>Findings</th><th>Risk & Action Plan</th><th>Screening Tips</th></tr>
<tr>
<td>Normal (no noted visual abnormalities)
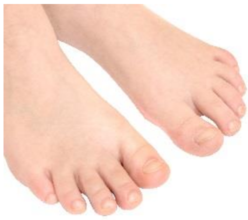</td>
<td>LOW RISK
Education
Provide low risk diabetes foot information
Follow-up
Foot exam/screen required annually</td>
<td>Inspect the general shape of both feet</td>
</tr>
<tr>
<td>Bunions
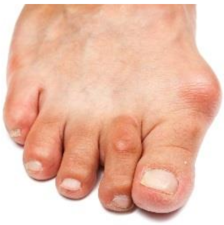</td>
<td rowspan="5">MODERATE RISK
Assessment
Identify decreased range of motion in ankle or toe joint
Identify structural abnormalities/deformities
Identify changes in structure (e.g. Charcot foot)
Identify previous amputations
Intervention: Referrals
High Risk Foot Team, AADL authorizer, other local healthcare providers such as an Orthotist, Podiatry
Patient should be seen within 1 month of assessment
Education
Provide moderate risk diabetes foot information
Follow-up
Foot assessment every 4-6 months</td>
<td rowspan="5">Most structural abnormalities are related to autonomic & motor neuropathy and further complicated by sensory neuropathy
Abnormal foot shape and prominent bony abnormalities can create pressure points that can lead to skin breakdown
All patients should be instructed in proper footwear to alleviate pressure on bony deformities & reduce skin breakdown (see Wounds Canada website for a handout on proper shoe fit: https://www.woundscanada.ca/about-dhfy
With loss of protective sensation (LOPS) patients may require therapeutic footwear and total contact inserts through Alberta Aids to Daily Living (AADL), see AADL website for current referral criteria:https://www.alberta.ca/alberta-aids-to-daily-living.aspx
Some insurance plans also offer assistance with payment if a prescription is provided
People with diabetes should inspect their footwear (look and feel) prior to putting shoes on
Appropriate footwear to be worn at all times, even in the house
Redistribution of pressure & modification of footwear is essential
Persistent corns and calluses due to structural deformities may warrant a surgical opinion regarding deformity correction
A chronic, stable Charcot foot follows an acute Charcot. The foot is no longer hot and structural deformities are stable</td>
</tr>
<tr><td>Claw or hammer toes
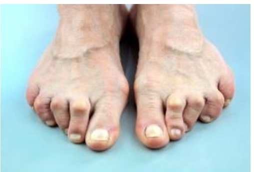</td></tr>
<tr><td>Overlapping digits
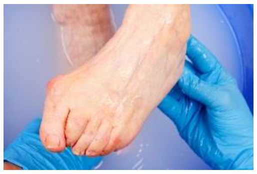</td></tr>
<tr><td>Reduced range of motion at the ankle or toe joints
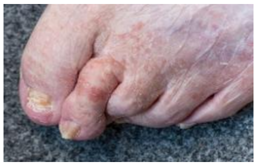</td></tr>
<tr><td>Arch deformities (high arch, fallen arch, rocker bottom, Charcot foot, etc.)
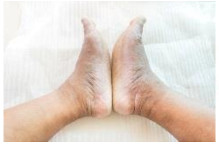 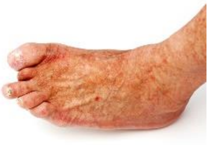</td></tr>
</table>

Structure/Anatomy Assessment continued

Findings	Risk & Action Plan	Screening Tips
**Partial or complete amputations of toes or foot**<br>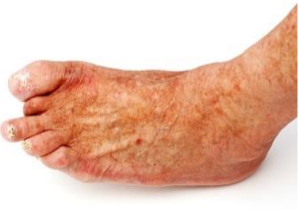	**MODERATE RISK CONTINUED**<br>**Assessment**<br>• Check footwear<br>• Identify any open areas, red areas, corns or calluses<br>**Intervention**<br>• Refer to HRFT or local specialist if there are open areas and LOPS and/or new footwear is required<br>• Patient should be seen within 1 month of assessment	• Footwear is often prescribed after amputation. The footwear may be wearing out. It is important to look at both the inside and outside of the footwear as offloading becomes very important when the foot anatomy has been altered
**Structural abnormalities with redness on pressure areas** (not infected)<br>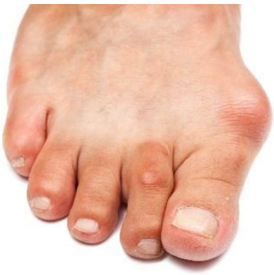	**HIGH RISK**<br>**Assessment**<br>• Redness over any structural deformities - pressure related<br>**Intervention**<br>• Refer to HRFT or local specialist<br>• Patient should be seen within 1-2 weeks of assessment<br>**Education**<br>• Provide high risk diabetes foot information<br>**Follow-up**<br>• Foot assessment **every 1-4 weeks**	• Reddened areas may progress to development of skin breakdown, wound(s) and/or infection in a short period of time. Note that early signs of infection are often subtle<br>• Pressure redistribution of plantar foot pressure & modification of offending footwear is essential
**Red, hot, painful joint**<br><br>**or**<br><br>**Acute Charcot joint "collapse"**<br>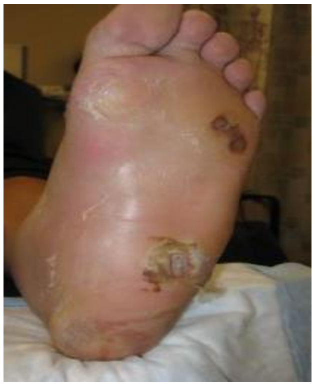	**URGENT RISK**<br>**Assessment**<br>• Red, hot, painful joint or acute Charcot foot<br>**Intervention**<br>• Antibiotic therapy<br>• Pain management**,** total offloading/non-weight bearing of foot is essential<br>• Refer for immediate treatment within 24 hours<br>• Referrals may include Orthopaedic Specialist, Infectious Disease (ID) Specialist<br>• May require hospital admission; if no admission required, needs close medical monitoring	• Treatment of acute Charcot foot requires immobilization of the foot, typically for several months & up to one year in a removable walker device or total contact cast (until excessive foot temperatures return to normal)<br>• Patient to completely offload pressure on affected foot. Damage done by walking on an acute Charcot foot is permanent<br>• Patient safety: if the patient is unable to safely offload the foot then provide appropriate aids such as a wheelchair<br>• Antibiotic therapy required guided by Diabetic Foot Infection Guidelines in BUGS & DRUGS or alternate resource

Sensation Testing

<table>
<tr><th>Findings</th><th>Risk & Action Plan</th><th>Screening Tips</th></tr>
<tr>
<td>Normal sensation to 10g monofilament exam
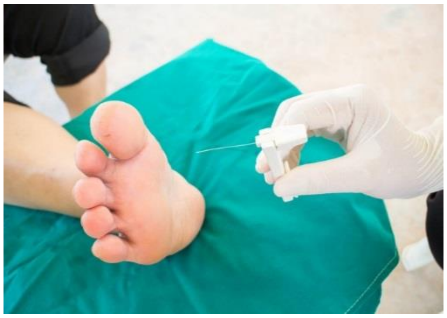</td>
<td>LOW RISK
Assessment
Assess for sensation using the 10 g Semmes-Weinstein 5.07 monofilament
Education
Provide low risk diabetes foot information
Follow-up
Foot exam/screen required annually</td>
<td rowspan="3">Sensory neuropathy or loss of protective sensation is a progressive problem affecting 40-50% of people with diabetes within 10 years of their diagnosis
Diabetic neuropathic pain is estimated to affect 20-24% of people with diabetes. This may be difficult to medically manage; however, there are many prescription medications that have been shown to reduce neuropathic pain
Loss of the ability to detect pain and temperature poses tremendous risk for puncture, pressure, friction, chemical and thermal injuries
For guidance on completing a monofilament test see next page</td>
</tr>
<tr>
<td>Patient sensation of the following:
Numbness/tingling/crawling or burning
Other patient descriptors to describe peripheral diabetic neuropathic pain may include:
Painful cold
Electric shocks
Pins and Needles
Itching</td>
<td rowspan="2">MODERATE RISK
Assessment
Ask the patient to describe any sensation or feeling they have in their legs/feet
Complete monofilament testing
Intervention
Intact sensation, refer to appropriate foot care provider if patient cannot perform self-foot care
Loss of sensation at one or more sites, refer to HRFT or local specialist
Patient to be seen within1 month of referral
Education
Provide patient with moderate risk information
Follow-up
Foot assessment every 4-6 months</td>
</tr>
<tr>
<td>Absent sensation using 10 g monofilament at 1 or more sites</td>
</tr>
<tr>
<td>Pain or inflammation in a previously insensate foot
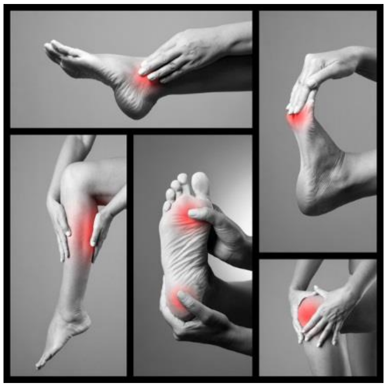</td>
<td>URGENT RISK
Assessment
Acute onset of pain in a previously insensate foot; may present with edema
Intervention
Total offloading/non-weight bearing of foot is essential
Refer for immediate treatment within 24 hours
Consult if appropriate
Infectious Disease (ID) Orthopaedic Specialist</td>
<td>Pain or inflammation may be related to infection, septic arthritis or an acute Charcot foot. This must be appropriately diagnosed and medically managed.
Antibiotic therapy required guided by Diabetic Foot Infection Guidelines in BUGS & DRUGS or alternate resource
Needs close medical monitoring
May require hospital admission</td>
</tr>
</table>

Sensation Testing continued

Monofilament testing is an inexpensive, easy-to-use, and portable test for assessing the loss of protective sensation, and it is recommended by several practice guidelines to detect peripheral neuropathy in otherwise normal feet.

Points of Emphasis for Monofilament Testing

- Use a 10-g Semmes-Weinstein 5.07 monofilament
- Should be done at least once a year as part of an overall foot screening and assessment
- Test both feet
- Conducted with patients who have any of the following:
  - Diabetes
  - Diabetic foot ulcer
  - Feelings of numbness, tingling, burning or a “crawling” sensation in one or both feet
- Screens for the presence or absence of neuropathy (sensation)
- Identifies Loss of Protective Sensation (LOPS)
  - LOPS is a major risk factor for developing diabetic foot ulcers which can lead to amputation
  - A positive screen is when sensation is absent at one or more of the 5 test sites
  - Feet may be falsely insensate when cold, edematous or heavily calloused
- Apply the monofilament to the skin and hold steady for several seconds
  - Approach, skin contact and departure of the filament should be approximately 1½ - 2 seconds duration
- Do not use a pen cap or pin for monofilament testing.

How to Perform Monofilament Testing

1. Provide a quiet and relaxed setting
2. Have patient remove shoes and socks on both feet – assist as necessary
3. Explain the procedure & show the patient the monofilament – understanding can enhance test results
4. Wash hands and apply gloves if needed – clean gloves are to be worn if there is an open area, discharge or a rash on the foot or ankle area
5. Touch the monofilament to the arm or hand so patient knows what to expect – what it feels like
6. Have the client close their eyes and indicate when they feel the monofilament touch by responding with a “yes” – also ask where they feel the monofilament
7. Hold the monofilament perpendicular to the foot and with a smooth, steady motion, touch the skin until the monofilament bends approximately 1 cm, applying sufficient force to bend it to a “C.” Hold it against the skin for approximately 2 seconds
8. Randomly test 5 sites on each foot – see diagram for site selection
9. Avoid any ulcers, calluses, sores, or scars – If an ulcer, callus or scar is on the foot, apply the monofilament on an area adjacent to rather than directly over the affected area
10. Revisit any sites where the patient did not respond to touch to confirm loss of sensation
11. Share the results of the test with the patient – provides a “teachable moment” to reinforce the concept and value of self-care

Hold the filament perpendicular to the skin and use a smooth motion when testing. Use a 3 step sequence that includes:

- Touch the skin
- Bend the filament
- Remove from the skin

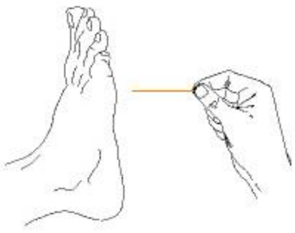

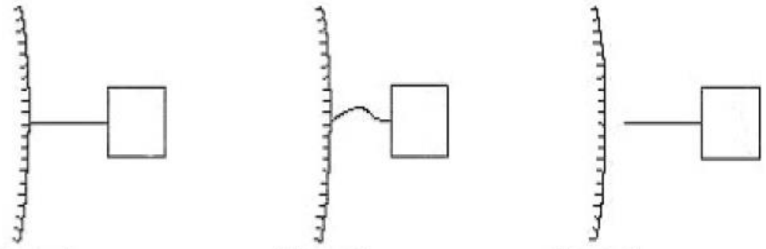

Figure 1 Figure 2 Figure 3

Figure adapted from British Columbia Provincial Nursing Skin & Wound Committee Resource

5-Site Monofilament Testing

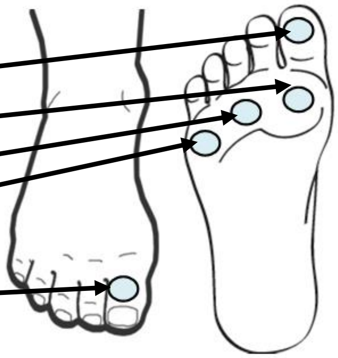

1. Plantar surface of the great toe
2. Plantar surface of the first metatarsal head
3. Plantar surface of the third metatarsal head
4. Plantar surface of the fifth metatarsal head
5. Dorsum of big toe (not on the toenail)

Interpretation of Results

- If the patient feels all 5 sites tested then the score is 0/5 and the patient has sensation
- If the monofilament is not felt in a tested area on the foot, this indicates loss of protective sensation (LOPS) in that area and should prompt a referral to a High Risk Foot Team or other appropriate community resource

Frequency of Testing

- Repeat testing should be done at least once a year and,
- When a foot ulcer occurs

Caring for the Monofilament

- Clean the filament as per the manufacturer's suggestions
- Ensure it is dry before storing
- Replace the monofilament if bowed, kinked, or twisted

Note:

For the purposes of the Diabetes Foot Screening Tool, a 5-site monofilament test has been adopted. This combines recommendations by the Canadian Diabetes Association and the Registered Nurses of Ontario. A 10-site assessment may also be used; however the literature indicates that the benefit of a 10 point test compared to a 5 point test is insignificant in predicting foot ulcer development.

Vascular Assessment – Identify PAD (Peripheral Arterial Disease)

Findings	Risk & Action Plan	Screening Tips
**Normal pulses**<br>**Normal capillary refill**<br>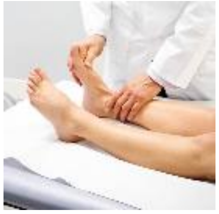<br>Posterior Tibialis<br>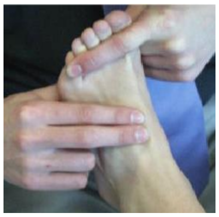<br>Dorsalis Pedis	**LOW RISK**<br>**Assessment**<br>• Palpate dorsalis pedis & posterior tibial pulses<br>(You can use a hand held Doppler to listen to pulses if available)<br>• Check capillary refill<br>• Check temperature of the skin on lower legs and feet<br>**Education**<br>• Provide low risk diabetes foot information<br>**Follow-up**<br>• Foot exam/screen required annually	• Pulses may be difficult to palpate in the presence of edema or if the feet are cold<br>• Check capillary refill by pressing against end of patient's toe until skin pales, then release. If color takes longer than 3-4 seconds to return, refill is delayed suggestive of arterial compromise
**Signs of peripheral arterial disease**<br>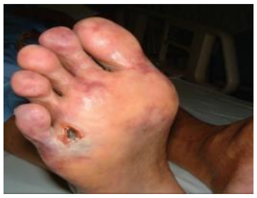<br>Cool skin with pallor, cyanosis or mottling (at rest or with leg elevation)<br>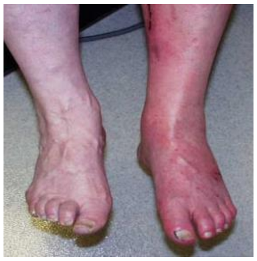<br>Dependent Rubor<br>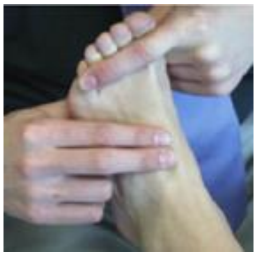<br>One or more pulses not palpable (dorsalis pedis & post-tibialis)	**HIGH RISK**<br>**Assessment**<br>• Assess the limbs for signs of ischemia<br>• Are one or both limbs cool? Have their legs and feet always been cool or is this a new condition?<br>• Does the limb blanch when elevated and become dark purple when dependent?<br>**Intervention**<br>• Refer to HRFT or local provider that can perform lower limb assessments<br>• Patient should be seen within 1-2 weeks of assessment<br>• Pressure redistribution of plantar foot pressure & modification of offending footwear is essential<br>**Education**<br>• Provide high risk diabetes foot information<br>**Follow-up**<br>• Foot assessment **every 1-4 weeks**	• **Decreased Skin Temperature** – May indicate arterial compromise especially if one foot is cooler than the other<br>• Ask what patient's normal over their lifetime has been. If cold outside, allow feet to warm up before your assessment<br>• Pallor on elevation of leg(s) above the level of the heart indicates that arterial perfusion is impaired<br>• Other signs of peripheral arterial disease include:<br>○ Thin, fragile, shiny skin<br>○ Loss of hair growth on lower leg (inadequate blood supply results in hair root death)<br>• **Dependent Rubor** – Skin has a purple color when leg is down<br>• Dependent rubor occurs when the leg is brought down below the level of the heart and blood rushes to the leg with the assistance of gravity. It is an indicator of poor arterial circulation<br>• **Pulses** – If edema prevents palpation of pulses, a Doppler could be used to assess presence or absence by auscultation. If there is no Doppler, referral for a lower limb assessment is needed

Vascular Assessment continued

Findings	Risk & Action Plan	Screening Tips
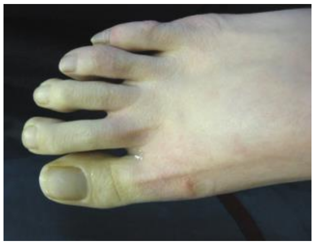<br>Absent pedal pulses with cold, white, painful foot or toes	**URGENT RISK**<br>**Assessment**<br>• Determine cause if possible<br>• Assess pain<br>**Intervention**<br>• Provide pain management<br>• Refer for immediate treatment within 24 hours<br>• Acute Care – may require hospital admission<br>• Vascular Surgeon	• Critical ischemia or significant loss of arterial perfusion to leg can be extremely painful even at rest<br>• Patient may be a candidate for urgent revascularization<br>• If not hospitalized, patient needs close medical monitoring<br>• Pressure redistribution is important to prevent alterations of the skin related to pressure, friction and/or shearing. Healing is often difficult or may not occur at all in the presence of PAD; pending the degree of compromise. Prevention is key

Additional Information

- Assessment of peripheral arterial circulation and identification of PAD is important in developing a comprehensive and holistic client centered health management plan
- Become familiar with signs of PAD (see Wounds Canada website: https://www.woundscanada.ca/)
- Clinical assessment includes identifying the presence of pedal pulses and claudication
  - Intermittent claudication can be an early warning sign of the presence of PAD and is progressive over time. Claudication can present as:
    - Leg muscle pain
    - Fatigue with walking (relieved by rest in minutes)
    - Pain at night when legs elevated
  - Assessment of pedal pulses with a Doppler (if available) is helpful if pedal pulses cannot be palpated; the screening team must have the skillset & equipment to perform this exam
- Advanced vascular assessment requires a referral: High Risk Foot Team, Lower Limb Clinic or Vascular Surgeon
- Lower Limb Assessment (LLA)
  - It is not expected that screening healthcare providers will complete this assessment
  - LLA requires training and skillset in order to make the clinical decisions in planning care
  - An Ankle Brachial Pressure Index (ABPI) is only one part of a lower limb assessment and decisions based solely on the ABPI value are not always clinically sufficient
- The Ankle Brachial Pressure Index (ABPI)
  - Non-invasive comparison of systolic pressures between the arm and the ankle identifying degree of arterial compromise is usually tested by a HRFT
  - Used as a screening tool to determine degree of arterial insufficiency and to identify individuals who require additional assessment/evaluation
  - It is important to recognize that:
    - Persons with diabetes may have a “false high” ankle brachial pressure index due to calcification of the vessels (greater than 1.3 mmHg: CDA, 2008)
    - ABPI is not useful for individuals with non-compressible vessels due to calcification or those patients with significant edema
    - Toe pressures using a PPG is recommended in these cases

Vascular Assessment continued

- It is recommended that individuals with diabetes undergo additional assessment such as toe pressures (PPG/photoplethysmography) or toe brachial index (TBI) to give additional information regarding peripheral arterial perfusion.
  - PPGs and TBIs are based on measurement of small vessel perfusion to the toes
- If edema is present, the history and cause of edema must be determined.
  - ***It is important that assessment of peripheral arterial circulation is completed prior to implementing an edema management plan, such as compression***

Ankle Brachial Pressure Index (ABPI)	Toe Pressure (PPG)	Toe Brachial Pressure Index	Ankle Doppler Wave Form	Diagnosis
**≥0.8-1.3 mm Hg**	≥50 mm Hg	≥0.7 mm Hg	Biphasic or Triphasic (Normal)	No significant arterial disease
**≥0.6- 0.8 mm Hg**	≥40 mm Hg	≥0.4-0.7 mm Hg	Biphasic / Monophasic	Arterial disease
**≥0.4-0.6 mm Hg**	<40 mm Hg	< 0.4 mm Hg	Monophasic	Significant arterial disease
**< 0.4 mm Hg**	≤25 mm Hg	≤ 0.2 mm Hg	Monophasic	High risk of critical limb ischemia

Note:

- These values may not be universally accepted as marginal variations exist within the current literature
- Accessibility to testing and interpretation of results may be limited to certain areas of specialty

Footwear Assessment

Findings	Risk & Action Plan	Screening Tips
**Footwear is appropriate and accommodates foot shape**<br>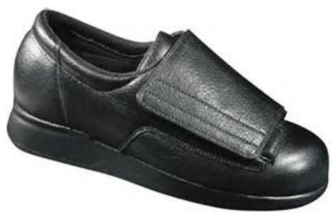	**LOW RISK**<br>**Assessment:**<br>• Visually and manually examine footwear inside and out at each screening visit<br>• Inspect feet for reddened areas that may indicate pressure points created by poorly fitted footwear<br>• Inspect socks for signs of blood or other discharge<br>**Intervention**<br>• Encourage patient to purchase "Diabetic Socks"<br>• Encourage the patient to be professionally fitted for appropriate footwear<br>**Education**<br>• At each visit, teach how to inspect footwear<br>• Shoes should be worn all the time when walking, even in the house<br>• Bare feet should be avoided<br>**Follow-up**<br>• Foot exam/screen required annually	• Footwear is the number one cause of trauma to the foot in people with diabetes<br>• Due to developing neuropathy, many patients are unable to feel shoes that are too small or too tight and will purchase shoes with poor fit<br>• Remember to ask patient how old their shoes are, what their regular footwear is and how often they wear their shoes (e.g. only outside or all of the time, even in the house)<br>• Seams in socks can cause pressure. Diabetic Socks have no seams and minimizes pressure areas on the feet<br>• Ask if the patient regularly wears socks<br>• Is footwear inappropriate (e.g. worn out, too tight, does not accommodate the foot shape)<br>• Cross reference with LOPS. If the patient has **intact sensation refer to appropriate foot care provider**<br>• If LOPS is present the patient may require therapeutic footwear
**Inappropriate footwear**<br>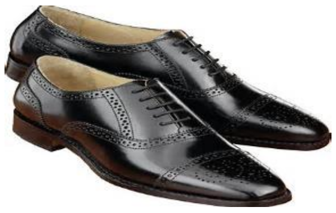<br>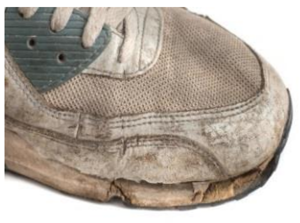	**MODERATE RISK**<br>**Assessment**<br>Check to see if shoes:<br>• Are too small, tight or loose<br>• Accommodate foot deformities<br>• Are worn-out<br>• Are "over the counter" or professionally fitted shoes<br>• Have rough seams or foreign objects inside the shoe<br>• Have abnormal wear patterns<br>Remove and inspect insoles<br>**Intervention**<br>• Requires new footwear<br>• If sensation is intact, refer to an Orthotist for custom orthotics<br>• Refer to AADL authorizer for therapeutic foot wear if no sensation (LOPS)	• Pressure redistribution of plantar foot pressure & modification of offending footwear is essential

Footwear Assessment continued

	**Education**<br>• Importance of appropriate footwear and self-assessment of feet<br>**Follow-up**<br>• Foot assessment **every 4-6 months**	
**Footwear causing pressure or skin breakdown**	**HIGH RISK**<br>**Assessment**<br>• Inspect foot including toes and heel for red areas or open skin/wounds<br>• Refer to sensation testing findings<br>**Intervention**<br>• Refer to HRFT or local specialist for AADL Therapeutic Footwear<br>• Patient should be seen within 1-2 weeks of assessment<br>**Education**<br>• Provide high risk diabetes foot information<br>**Follow-up**<br>• Frequent foot assessments should occur once appropriate foot wear has been obtained	• With loss of sensation at one or more sites the patient may require therapeutic footwear<br>• Off the shelf orthotics are not sufficient to meet the needs of patients who are high risk for developing a foot ulcer related to pressure from footwear<br>• AADL will provide footwear for persons with diabetes with a cost sharing component. See AADL website for most current criteria https://www.alberta.ca/alberta-aids-to-daily-living.aspx

Helpful Hints

- Consider referring the patient for professionally fitted footwear if “off the shelf” options do not accommodate foot challenges or support the offloading of pressure areas.
- **It is important once the patient has LOPS that footwear be professionally fitted**
- Finding the Proper Shoe Fit: https://www.woundscanada.ca/86-diabetic-healthy-feet-and-you/for-patients-and-public/diabetes-healthy-feet-and-you/steps-for-healthy-feet/278-steps-for-healthy-feet-2

Proper Foot Care

Causes of diabetes foot problems and ulcerations include poor foot hygiene, inability to perform self-care, infrequent/improper inspection of feet and inappropriate or poorly fitting footwear.

Healthcare providers play a key role as patient advocates, enabling patient accessibility to proper foot care and footwear.

Along with proper footwear, it is essential that the patient appreciate the importance of proper foot care. A patient education booklet has been developed to support patient self-management and to help recognize when they should see their healthcare provider. This booklet is called “**Foot Care for People with Diabetes: Low, Moderate and High Risk Care Recommendations”** and can be found on the MyHealthAlberta website at https://myhealth.alberta.ca/Alberta/Pages/foot-care-for-people-with-diabetes.aspx

Feel free to provide the patient with this resource.

The findings below are not part of the screening however, it provides healthcare providers an opportunity to review the patient resource material

Proper Foot Care continued

Findings	Risk & Action Plan	Screening Tips
**Adequate foot care**<br>(healthy skin, nails)<br>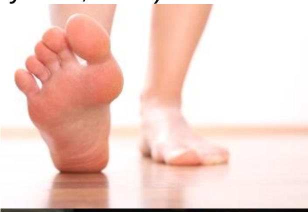<br>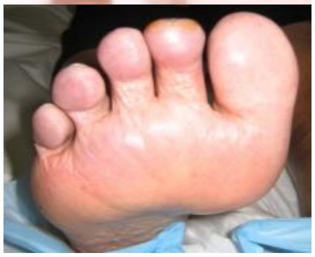	**LOW RISK**<br>**Assessment**<br>• Inspect the feet for signs of poor foot hygiene (dirty, long or poorly shaped nails, calloused or cracked skin)<br>**Intervention**<br>• Reinforce need for proper foot care and assess for potential barriers to proper foot care<br>**Follow-up**<br>• Foot exam/screen required annually	• Ask if foot care assistance is required for hygiene and for performing daily foot inspections<br>• If assistance is required, determine what assistance is needed (poor vision, range of motion, self-care, mobility, etc.)<br>• Toenail care must be done properly to prevent injury to the toenail and/or toe<br>• Discuss and arrange assistance for foot care as needed (family, friend, foot care provider)<br>• Identify foot care providers in the area and their costs, and provide the patient with a list
**Inadequate foot care**<br>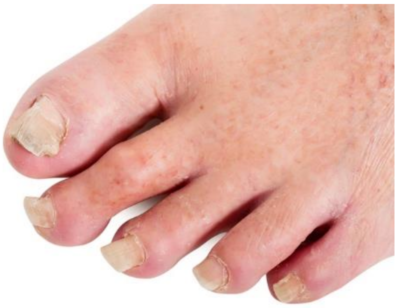	**MODERATE RISK**<br>**Assessment**<br>• Discuss the need for professional nail care<br>• May require information on moisturizers<br>**Intervention**<br>• Provide a list of foot care providers and potential costs<br>• Recommend nail care to be done within 1 month<br>**Follow-up**<br>• Foot assessment every **4-6 months**	• A moderate risk patient should be receiving professional nail care<br>• Patients with dry skin and poor foot hygiene will require medical grade moisturizers<br>• Identify any barriers to foot and nail care<br>• The patient can be shown how to inspect their feet with a mirror
**Grossly abnormal skin or nails, specialty care**<br>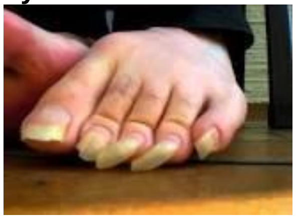<br>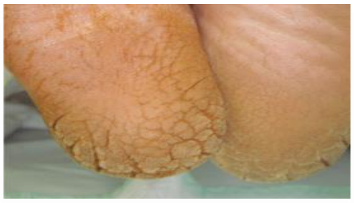	**HIGH RISK**<br>**Assessment**<br>• Identify the abnormal condition<br>**Intervention**<br>• Refer to HRFT or local provider (e.g. Podiatrist)<br>• Provide high risk diabetes foot information<br>**Follow Up**<br>• Foot assessment **every 1-4 weeks**	• Determine if the patient has been inspecting their feet<br>• Identify any over the counter treatment they may have been doing to address the abnormal condition<br>• Reinforce the need for daily foot inspections and when it would be appropriate to see their physician for foot and nail related changes

Notes:

Notes:

Alberta Health Services

Affix patient label within this box

Diabetes Foot Screening Tool

EXAM	FINDINGS	R	L	RISK
SKIN	Normal intact skin – healthy or dry *check in between toes			LOW
	Callus/Corn/Fissure/Crack not bleeding or draining			MODERATE
	Prior history of Diabetic Foot Ulcer(s) ulcer in remission			
	Blister = B or Hemorrhagic callus = HC			HIGH
	Fissure or Crack Bleeding or draining = F			
	Diabetic Foot Ulcer – Not infected and/or with intact dry black eschar = U			
	**Infected Diabetic Foot Ulcer or wet gangrene**			**URGENT**
NAILS	Normal well-kept with minimal discoloration			LOW
	Missing, sharp, unkept, thickened, long or deformed			MODERATE
	Infected ingrown nail			
STRUCTURE ANATOMY	Normal no noted visual abnormalities			LOW
	Decreased range of motion at ankle or toe joint			MODERATE
	Deformities Bunion/Hammer or claw toes/overlapping toes			
	Structure Fallen Arch/ Rocker bottom foot/stable Charcot foot			
	Previous amputation **X** over location or draw/describe on diagram			
	Redness over any structural deformities pressure related			HIGH
	**Red, hot painful joint or acute Charcot foot**			**URGENT**
SENSATION Testing for LOPS	Normal sensation using 10 g monofilament at the 5 predetermined sites			LOW
	Sensation of numbness/tingling/throbbing/burning			MODERATE
	Absent or altered sensation at one or more of the five sites			
	**Acute onset of pain in a previously insensate foot**			**URGENT**
VASCULAR Testing for Arterial Compromise	Normal pulses normal capillary refill			LOW
	Signs of Ischemia (PAD)<br>Cool skin with pallor, cyanosis or mottling, and/or dependent rubor			HIGH
	One or more pulses not palpable or audible (Doppler)			
	**Absent pedal pulses with cold white painful foot or toes**			**URGENT**
FOOTWEAR	Appropriate accommodates foot shape			LOW
	Inadequate Footwear			MODERATE
	Inappropriate Footwear causing pressure/skin breakdown			HIGH

Instructions: *Refer to Health Provider's Guide to Diabetes Foot Screening*
Mark ulceration location (U). Mark other areas of specific concern: blister (B), draining fissure/crack (F), hemorrhagic callus (HC), and previous amputation (X).

Sensation Testing (monofilament)

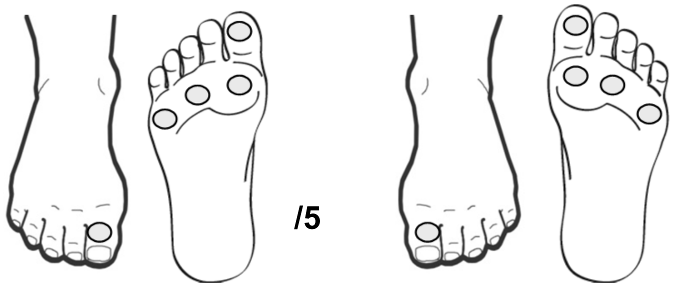

/5 **/5**

Fill in if no sensation ●

Leave blank if sensation present ○

RIGHT **LEFT**

Identify any wounds and location on the foot or toe(s)

Date	Signature	Primary Care Site

Comments

20710(Rev2017-03)

Diabetes Foot Care Referral Process Guidelines

Alberta Health Services

Risk score: determined by the highest risk score that is assessed in any category during the foot screening
All patients, regardless of risk score, should be counselled on healthy diet and exercise and treated to achieve and maintain target A1C, BP, cholesterol, and tobacco cessation. Patients who are exposed to tobacco should be encourage to contact Albertaquits.ca

LOW RISK

Normal foot exam

- Provide **Low Risk** Information
- Inform where to access local resources

↓

Foot Care Management and follow up by:
- Primary Care Provider

(Care by a foot specialist is not required for the Low Risk Diabetes Foot)

↓

Foot Assessment
Annually

MODERATE RISK

Skin, nail, anatomical or sensory abnormality with no skin breakdown/ulcer

- Provide **Moderate Risk** Informationon
- Inform where to access local resources

↓

Referral to family physician (FP) or nurse practitioner (NP) **within 1 month** for management or referral to High Risk Foot Team* If appropriate

Findings	***Foot Specialist***
Skin/nail abnormalities	*Foot care provider***
Structural Deformities or Inadequate/ Inappropriate footwear	*Occupational therapist (OT)*<br>*Orthotist*<br>*Pedorthist*
Vascular concerns	*FP/NP/Vascular assessment*
Loss of protective sensation (LOPS)	*Diabetes and foot care education*<br>*Orthotist*<br>*Pedorthist*
Neuropathic pain	*FP/NP*

↓

Foot Screen Assessment
Every 4 to 6 months
(or as assessed by above)

HIGH RISK

Skin breakdown/ulcer, impaired circulation with no signs of infection or cellulitis

- Provide **High Risk** Information
- Inform where to access local resources

↓

Refer to e High Risk Foot Team* or local team **within 1 to 2 weeks.** Note: all cases or ulcers and structural deformities + pressure require offloading. Air casts, non weight bearing with crutches, etc. may provide interim relief, but patients will need referral to a Orthotist, OT or other specialist in footwear modification.

Findings	***Foot Specialist***
Skin (non-infected ulcer/skin breakdown/ hemorrhagic callous)	*FP/NP-wound management + offloading*
Structural deformity with pressure	*FP/NP +/-*<br>*Occupational therapist (OT)*<br>*Orthopedic specialist*<br>*Podiatric physician*<br>*+ offloading*
Vascular Concerns	*FP/NP +/-*<br>*Vascular surgeon*

↓

Foot Screen Assessment
Every 1 to 4 weeks

URGENT

Cellulitis, draining ulcer, acute charcot joint collapse, gangrene, cold white painful foot or part thereof

Contact MD/FP/NP/ER or Urgent Care Services to arrange for **immediate (within 24 hours) assessment and treatment.**
Offload affected area

↓

Findings	***Foot Specialist***
Skin/wound Infection	*FP/NP +/-*<br>*Infectious Disease (ID) or ER or Wound Care Specialist*
Structural Deformities (red hot painful joint or acute Charcot joint "collapse")	*Orthopedic specialist*<br>*Occupational therapist (OT)*<br>*Orthotist*<br>*Pedorthist*<br>*Podiatric physician*
Vascular concerns (gangrene or cold white painful foot/ toes)	*Vascular surgeon/ Interventional radiologist*
Pain or inflammation in a previously insensate foot	*FP/NP or ER*

↓

Arrange for follow-up education once stable

CLOSED ULCER (Ulcer in Remission)

After ulcer closure, patients remain at significant risk for recurrence of ulceration. The High Risk Foot Team will follow these patients until appropriate for transition back to primary care for long term follow-up.

(High Risk and Urgent pathways lead to Closed Ulcer; Closed Ulcer leads back to Moderate Risk Foot Screen Assessment.)

***Referral to a High Risk Foot Team may include interventions from several disciplines. If there is not a high risk foot team in your area refer patients to local resources for care.**
**** Foot care provider may include podiatric physician, foot care nurse, or other medically trained provider competent in providing skin and nail care. These services may have a fee.**

AHS Diabetes, Obesity & Nutrition Strategic Clinical Network™ July 2017
Adapted from the New Brunswick Diabetes Foot Care Clinical Pathway

Alberta Health Services

Affix patient label within this box

Diabetes Foot Risk Assessment Triage Referral

Date of Screening and Triage *(yyyy-Mon-dd)*	HRFT Fax #

- Send the completed Diabetic Foot Screening Tool and Triage Referral Form with your site based referral form to the High Risk Foot Team.
- The High Risk Foot Team will follow referred patients until foot risk factors are addressed - appropriate interventions initiated. Transition of ongoing foot management plan will be communicated to referring Primary Care site.

Risk Features *(check all that apply)* (✔)

Low Risk ► Routine annual foot exam & diabetes education **Managed by Primary Care**

Moderate Risk Criteria with or without Loss of Protective Sensation

☐ Callus/Corn/Fissure/Crack *(not bleeding or draining)*
☐ Inadequate foot care - missing, sharp, unkept, thickened, long or deformed toe nails
☐ Inadequate foot wear ☐ Infected ingrown toe nail
☐ Sensation of numbness/tingling/throbbing/burning

► **Refer to Foot Care Provider: podiatrist or trained foot care nurse**
► **Foot exam every 4-6 months or as per assessed need** **Managed by Primary Care**

Moderate Risk Criteria - Loss of Protective Sensation at one or more of 5 identifed sites, **PLUS** any of the following:

☐ Prior history of Diabetic Foot Ulcer *(ulcer in remission)* and or amputation
☐ Decreased range of motion at ankle or toe joint ☐ Foot Deformities
☐ Inadequate footwear requiring therapeutic/custom footwear ☐ Altered structure

► **Refer to High Risk Foot Team or local health care professional**
(recommended patient be seen within one month of referral) **Managed by High Risk Foot Team**

High Risk Criteria - Patient presents with **one or more** of the following:

☐ Blister, fissure or crack *(bleeding or draining)* and or hemorrhagic callus
☐ Diabetic Foot Ulcer
☐ Redness over structural deformity of the foot /toes related to pressure
☐ Signs of arterial insufficiency *(PAD; ischemia)* cool skin with pallor, cyanosis or mottling, dependent rubor
☐ One or more pedal pulses not palpable or audible
☐ Inappropriate footwear causing pressure and/or skin breakdown

Refer to:
► **High Risk Foot Team or local health care professional(s)** *(recommend patient be seen within 2 weeks of referral)*
► **Infectious Disease** for consultation if warranted
► **Vascular Surgeon** if appropriate
► **Antibiotic therapy** *(Guided by Diabetic Foot Infection Guidelines in BUGS AND DRUGS 2012 or consult Infectious Disease)* **Managed by High Risk Foot Team**

Urgent Risk Criteria - Patient presents with **one or more** of the following :

☐ Infection - draining Diabetic Foot Ulcer and /or wet gangrene
☐ Red, hot, painful joint, or acute Charcot foot
☐ Acute onset of pain in a previously insensate foot
☐ Absent pedal pulses with cold white painful foot or toes

► **Primary Provider Initiates antibiotic therapy** guided by Diabetic Foot Infection Guidelines in BUGS AND DRUGS 2012 and/or consult Infectious Disease
► **Offload the affected foot**
► **Refer to the appropriate health care provider** based on the patient assessment findings *(ie Foot and Ankle Surgeon, or Vascular Surgeon if absent pedal pulses on auscultation)*
► **May Require Acute Care Admission**
► **Refer to High Risk Foot Clinic once patient is stable and specialist referrals have been arranged**

Comments

Date Faxed *(yyyy-Mon-dd)*	High Risk Foot Team	Signature

Diabetes

Diabetic Foot Care for the Low Risk Foot

When you have diabetes you should ask your healthcare provider to examine your feet at least once a year.

Your foot exam shows you are at **low risk** for having foot problems. You have a normal foot, with no open sores, normal feeling, and no changes to your bones.

How can I protect my feet?

1. **Check your feet daily.** Look between your toes and at the top and bottom of your feet for swelling, redness, sores, corns, or calluses. Use a mirror or ask someone to help if you can't see your feet.
2. **Wash your feet daily** with warm water and mild soap. Make sure you dry well between your toes.
3. **Cut your toenails straight across** (not on an angle) and smooth the edges with a nail file.
4. **Apply a moisturizer (lotion)** at the top and bottom of your feet every day. Do NOT use moisturizer between your toes. Use a moisturizer containing urea. Ask your healthcare provider for examples.
5. **Have your feet checked once a year** by your healthcare provider.
6. **Wear shoes that fit well.**
7. **If you smoke, try to quit or cut down.** If you want help, call Alberta Quits at **1-866-710-QUIT (7848)**.

Warning Signs:

- pain in your calves on walking that doesn't go away when you rest
- burning sensation or new pain in your feet
- red areas over a toe or toes
- getting corns, calluses, or blisters

Report any of these changes to your healthcare provider as soon as possible (within a week). Ask if you need more testing or a referral to another specialist

Call your healthcare provider **right away** and ask to be seen the same day, or go to the emergency department if you have any of these:

- cold, painful, discoloured (pale, blue, or red) feet
- red, hot, swollen feet that may or may not be painful
- new or increasing pain in your lower legs or feet
- any open sore on your foot that is draining

Steps toward good foot health

(Reproduced with permission from Diabetes Canada 2018 Clinical Practice Guidelines)

Do:

- Do wear shoes that fit well. Shoes should have good support, do not rub or pinch your feet, and have low heels (less than 5 cm or 2 in). Consider having your shoes "professionally fitted".
- Do buy your shoes late in the day. Your feet may swell slightly during the day.
- Do wear socks at night if your feet get cold.
- Do wiggle your toes and move your ankles for 5 minutes, 2 or 3 times a day, to help blood flow in your feet and legs.
- Do put your feet up when you are sitting.
- Do activity every day to improve blood flow through your body.

Do NOT:

- Do not use over-the-counter medicines to treat warts or corns. They are not safe for people with diabetes.
- Do not wear anything tight around your legs such as tight socks or knee-highs. This is not good for your circulation.
- Do not go barefoot. Always wear shoes inside and outside your home.
- Do not use hot water bottles, heat bags, or heating pads on your feet.
- Do not cross your legs or sit for long periods of time.
- Do not use over-the-counter insoles unless recommended by your foot expert. They can cause blisters if they do not fit well.
- Do not smoke. Smoking decreases blood flow and healing. It also increases the risk of amputation.

Your self-care management plan

As you take care of your feet, you need to closely watch your blood sugar levels, blood pressure, and cholesterol. Keep track of your results to make it easier to review and plan your care. Together you and your healthcare provider will decide on the best levels for you.

Indicator	Present Level	Goal Level
Blood sugar or AIC		
Blood Pressure		
Cholesterol		

Adapted from the New Brunswick Diabetes Foot Care Clinical Pathway

For 24/7 nurse advice and general health information call Health Link at 811.

Current as of: April 15, 2020

Author: Diabetes, Obesity & Nutrition Strategic Clinical Network, Alberta Health Services

This material is for information purposes only. It should not be used in place of medical advice, instruction, or treatment. If you have questions, talk with your doctor or appropriate healthcare provider. This information may be printed and distributed without permission for non-profit, education purposes. The content on this page may not be changed without consent of the author. Contact feedback@myhealth.alberta.ca.

MyHealth.Alberta.ca

Diabetes

Diabetic Foot Care for the Moderate Risk Foot

When you have diabetes you should ask your healthcare provider to examine your feet at least once a year.

Your foot exam shows you are at **moderate risk** for having foot complications. Your healthcare provider will talk with you about any extra appointments you might need for your feet.

You are at moderate risk of having foot problems (complications) if you have any of these:

- problems with your skin or nails (e.g., redness over a toe or other area, ingrown toe nail)
- corns or calluses
- problems with sensation (e.g., a feeling of numbness or tingling in your legs and feet, or pain in your legs when you are walking that goes away when you rest)

If you see any of these problems, make an appointment to see your healthcare provider within 1 month. Your healthcare provider will talk with you about any extra appointments you might need for your feet.

How to Lower your Chances of Having Serious Foot Problems

1. **If you smoke, try to quit or cut down.** Smoking can cause less blood to flow to your foot making it harder for foot ulcers to heal. If you want help, call Alberta Quits at **1-866-710-QUIT (7848)**.
2. **Check your feet daily.** Look between your toes and at the top and bottom of your feet for swelling, redness, sores, corns, or calluses. Use a mirror to check the bottoms of your feet or ask someone to help you if you can't see your feet.
3. **Wash your feet daily** with warm water and mild soap. Make sure you dry well between your toes.
4. **Have someone who is properly trained** cut your toe nails.
5. **Apply a moisturizer (lotion)** at the top and bottom of your feet every day. Do NOT put moisturizer between your toes. Use a moisturizer containing urea. Ask your healthcare provider for examples.
6. **Wear shoes that fit well.** If you have lost feeling in your feet, have your shoes professionally fitted. Poorly fitting shoes can lead to pressure areas, corns, calluses, and blisters which can lead to sores on your feet or toes.

Warning Signs:

- pain in your calves on walking that doesn't go away when you rest
- burning sensation or new pain in your feet
- red areas over a toe or toes
- getting corns, calluses, or blisters

Report any of these changes to your healthcare provider (within a week). Ask if you need more testing or a referral to another specialist

Call your healthcare provider **right away** and **and request to be seen the same day**, or go to the emergency department if you have any of these:

- cold, painful, discoloured (pale, blue, or red) feet
- red, hot, swollen feet that may or may not be painful
- new or increasing pain in your lower legs or feet
- any open sore on your foot that is draining

Steps toward good foot health

(Reproduced with permission from Diabetes Canada 2018 Clinical Practice Guidelines)

Do:

- Do wear shoes that fit well. Shoes should have good support, do not rub or pinch your feet, and have low heels (less than 5 cm or 2 in). Consider having your shoes "professionally fitted".
- Do buy your shoes late in the day. Your feet may swell slightly during the day.
- Do wear socks at night if your feet get cold.
- Do wiggle your toes and move your ankles for 5 minutes, 2 or 3 times a day, to help blood flow in your feet and legs.
- Do put your feet up when you are sitting.
- Do activity every day to improve blood flow through your body.

Do NOT:

- Do not use over-the-counter medicines to treat warts or corns. They are not safe for people with diabetes.
- Do not wear anything tight around your legs such as tight socks or knee-highs. This is not good for your circulation.
- Do not go barefoot. Always wear shoes inside and outside your home.
- Do not use hot water bottles, heat bags, or heating pads on your feet.
- Do not cross your legs or sit for long periods of time.
- Do not use over-the-counter insoles unless recommended by your foot expert. They can cause blisters if they do not fit well.
- Do not smoke. Smoking decreases blood flow and healing. It also increases the risk of amputation.

Your self-care management plan

As you take care of your feet, you need to closely watch your blood sugar levels, blood pressure, and cholesterol. Keep track of your results to make it easier to review and plan your care. Together you and your healthcare provider will decide on the best levels for you.

Indicator	Present Level	Goal Level
Blood sugar or AIC		
Blood Pressure		
Cholesterol		

Adapted from the New Brunswick Diabetes Foot Care Clinical Pathway

For 24/7 nurse advice and general health information call Health Link at 811.

Current as of: April 15, 2020

Author: Diabetes, Obesity & Nutrition Strategic Clinical Network, Alberta Health Services

This material is for information purposes only. It should not be used in place of medical advice, instruction, or treatment. If you have questions, talk with your doctor or appropriate healthcare provider. This information may be printed and distributed without permission for non-profit, education purposes. The content on this page may not be changed without consent of the author. Contact feedback@myhealth.alberta.ca.

MyHealth.Alberta.ca

Diabetes

Diabetic Foot Care for the High Risk Foot

When you have diabetes you should ask your healthcare provider to examine your feet at least once a year.

Your foot exam shows you are at high risk for serious foot problems. Your healthcare provider will make a referral for you to see a foot care specialist. You should expect a phone call to book an appointment in 1 to 2 weeks. If you do not get an appointment, please call your healthcare provider to let them know you are still waiting for an appointment.

You are at high risk for serious foot problems (complications) if you have any of these:

- problems with your skin or nails (e.g., redness over a toe or other area, ingrown toe nail)
- corns or calluses
- problems with sensation (e.g., a feeling of numbness or tingling in your legs and feet, or pain in your legs when you are walking that goes away when you rest)

plus you have any of these 3:

1. loss of feeling (loss of protective sensation)
2. less blood flow to your feet (poor circulation)
3. have an open sore on your foot

If you see any of these problems, make an appointment to see your healthcare provider right away.

How to Lower Your Chances of Amputation

1. **Stay off your foot with the sore** (ulcer) as much as possible. You might need to use crutches or special footwear to keep weight off the sore.
2. **If you smoke, try to quit or cut down.** Smoking can cause less blood to flow to your foot making it harder for your foot to heal. If you want help call Alberta Quits at **1-866-710-QUIT (7848)**.
3. **Check your feet daily.** Look between your toes and at the top and bottom of your feet. Use a mirror or ask someone to help if you can't see your feet. Look for swelling, redness, new areas of breakdown, or changes to your sore. Check if there is more fluid leaking from the sore or there is a smell (odour). **If you see these changes call your healthcare provider right away.**
4. **Wash your feet daily and dry well especially between your toes.** Check the water temperature if you have lost protective sensation by using your elbow or a thermometer. The temperature should be between 32 to 35°C (90 to 95°F). If you have a wound dressing that covers your sore, follow the directions from your healthcare provider to properly clean your feet.
5. **Have someone who is properly trained** cut your toe nails.
6. **Apply a moisturizer (lotion)** at the top and bottom of your feet every day. Do NOT apply between your toes or on any open sores, unless you are told to do so by your doctor. Use a moisturizer containing urea. Ask your healthcare provider for examples.

Steps toward good foot health

(Reproduced with permission from Diabetes Canada 2018 Clinical Practice Guidelines)

Do:

- Do wear shoes that fit well. Shoes should have good support, do not rub or pinch your feet, and have low heels (less than 5 cm or 2 in). Consider having your shoes "professionally fitted".
- Do buy your shoes late in the day. Your feet may swell slightly during the day.
- Do wear socks at night if your feet get cold.

- Do wiggle your toes and move your ankles for 5 minutes, 2 or 3 times a day, to help blood flow in your feet and legs.
- Do put your feet up when you are sitting.
- Do activity every day to improve blood flow through your body.

Do NOT:

- Do not use over-the-counter medicines to treat warts or corns. They are not safe for people with diabetes.
- Do not wear anything tight around your legs such as tight socks or knee-highs. This is not good for your circulation.
- Do not go barefoot. Always wear shoes inside and outside your home.
- Do not use hot water bottles, heat bags, or heating pads on your feet.
- Do not cross your legs or sit for long periods of time.
- Do not use over-the-counter insoles unless recommended by your foot expert. They can cause blisters if they do not fit well.
- Do not smoke. Smoking decreases blood flow and healing. It also increases the risk of amputation.

Your self-care management plan

As you take care of your feet, you need to closely watch your blood sugar levels, blood pressure, and cholesterol. Keep track of your results to make it easier to review and plan your care. Together you and your healthcare provider will decide on the best levels for you.

Indicator	Present Level	Goal Level
Blood sugar or AIC		
Blood Pressure		
Cholesterol		

Warning Signs:

- pain in your calves on walking that doesn't go away when you rest
- burning sensation or new pain in your feet
- red areas over a toe or toes
- getting corns, callus or blisters

Report any of these changes to your healthcare provider as soon as possible (within a week). Ask if you need more testing or a referral to another specialist.

Call your healthcare provider right away and request to be seen the same day, or go to the emergency department if you have any of these:

- cold, painful, discoloured (pale, blue, or red) feet
- red, hot, swollen feet that may or may not be painful
- new or increasing pain in your lower legs or feet
- any open sore on your foot that is draining.

Adapted from the New Brunswick Diabetes Foot Care Clinical Pathway

For 24/7 nurse advice and general health information call Health Link at 811.

Current as of: April 15, 2020

Author: Diabetes, Obesity & Nutrition Strategic Clinical Network, Alberta Health Services

This material is for information purposes only. It should not be used in place of medical advice, instruction, or treatment. If you have questions, talk with your doctor or appropriate healthcare provider. This information may be printed and distributed without permission for non-profit, education purposes. The content on this page may not be changed without consent of the author. Contact feedback@myhealth.alberta.ca.
